# Effect of Farming System on Grain Composition and Immunogenic Potential of Ancient and Modern Durum Wheat Varieties

**DOI:** 10.3390/foods15071121

**Published:** 2026-03-24

**Authors:** Anis Boukrain, Cristina Martínez-Villaluenga, Juana Frias, Mondher Mejri, Elena Peñas

**Affiliations:** 1 Laboratory of Functional Physiology and Valorization of Bio-Resources (LR23ES08), Higher Institute of Biotechnology of Beja, University of Jendouba, Beja 9000, Tunisia; anisboukrain@outlook.com (A.B.); mondher.mejri@u-jendouba.tn (M.M.); 2 Institute of Food Science, Technology and Nutrition (ICTAN-CSIC), Jose Antonio Novais 6, 28040 Madrid, Spain; c.m.villaluenga@csic.es (C.M.-V.); frias@ictan.csic.es (J.F.)

**Keywords:** durum wheat, farming system, protein fractions, quality, immunogenicity, celiac disease, peptidomics

## Abstract

Organic farming is increasingly promoted as a sustainable alternative to conventional wheat production; however, its effects on grain quality and immunogenic potential remain insufficiently understood. This study evaluated the influence of the farming system (organic vs. conventional) on grain composition, technological quality traits, immunochemical reactivity, and immunogenic peptide profiles in 13 durum wheat varieties, including traditional and modern Tunisian varieties. Protein fraction content, amino acid composition, gluten-quality parameters, starch content, and immunochemical reactivity against anti-gliadin antibodies were determined. In addition, in vitro digestion followed by LC–MS/MS peptidomic analysis and epitope mapping was performed on representative ancient and modern varieties to investigate the release of celiac-disease-related immunogenic peptides after simulated gastrointestinal digestion. Protein content and quality traits were mainly genotype-dependent, with no consistent effect from the farming system across all varieties. Organic farming was associated with reduced starch accumulation (3.2–39.9% reduction) and lower immunochemical reactivity. Peptidomic analysis further revealed a reduced number and relative abundance (20.8–43.6% lower abundance) of immunogenic peptides in organically cultivated wheat compared with conventionally grown counterparts. This study highlights the significant interaction between the genotype and farming system, and provides a novel demonstration that organic management can reduce the abundance of celiac-disease-related immunogenic peptides, particularly in ancient varieties.

## 1. Introduction

Organic farming has gained increasing attention as a sustainable alternative to conventional agricultural systems, particularly in cereal production such as wheat. Previous studies have shown that organic management practices can improve soil fertility, enhance microbial activity, and promote long-term soil health, thereby supporting stable wheat production with reduced reliance on synthetic inputs [1]. In addition, organically grown wheat has been reported to achieve yields that are comparable to conventional systems while improving the grain’s nutritional quality, including higher levels of dietary fiber and essential micronutrients such as iron, zinc, and selenium [2]. From a broader food systems perspective, organic wheat farming contributes to environmental sustainability by reducing nutrient losses and increasing agroecosystem diversity, reinforcing its potential role in resilient and sustainable food production systems [3].

Tunisia has considerable potential for the development of organic durum wheat systems. Durum wheat is the dominant cereal crop in the country and diversification toward organic and low-input farming systems has been identified as a promising strategy to enhance food security while responding to the increasing demand for high-quality and value-added products [4,5]. At the same time, Tunisia faces the dual challenge of satisfying domestic demand and strengthening its position in export markets. The country possesses several strategic advantages for organic cereal production, including proximity to European markets, sustained demand for organic wheat, and the high technological and nutritional quality, particularly the elevated protein content, of certain traditional durum wheat varieties [6]. Nevertheless, the success of organic durum wheat production is strongly dependent on varietal choice [7]. Cultivars bred for high-input conventional systems often fail to express the optimal yield stability and quality under organic conditions [8]. Although varietal adaptation is recognized as a key determinant of both productivity and quality [9], systematic evaluations of durum wheat genotypes under organic conditions remain scarce, particularly in a North African agroecological context.

Wheat prolamins are responsible for triggering celiac disease, an autoimmune enteropathy which affects approximately 1% of the general population [10]. Immunogenicity has been shown to vary among wheat genotypes [11], which is primarily due to differences in the number and sequence of epitope regions within gliadins [12]. These proteins contain proline-and glutamine-rich sequences that give rise to digestion-resistant immunogenic peptides, such as the 33-mer α-gliadin peptide, which are recognized by HLA-DQ2- or HLA-DQ8-restricted CD4^+^ T lymphocytes, triggering the characteristic immune response of celiac disease [13,14]. While differences between ancient and modern wheat varieties have been reported, with conflicting evidence regarding their relative immunogenicity [15], the potential influence of farming systems on the abundance of immunoreactive motifs in durum wheat proteins has received far less attention. Recent findings suggest that agronomic practices may modulate protein expression and peptide profiles in wheat [16], but comprehensive comparative data integrating genotype and farming system effects are still lacking.

To date, only few studies have jointly addressed the interaction between the wheat genotype and farming system in grain composition (including protein fraction and starch) and technological quality traits in durum wheat [17,18]. This lack of integrative evidence limits the ability to identify varieties that combine agronomic performance, technological quality, and reduced immunogenic potential. Therefore, the aim of the present study was to evaluate the influence of conventional and organic farming systems on grain composition, quality-related traits, immunochemical reactivity and immunogenic peptide profile of 13 durum wheat varieties, including both local traditional and modern varieties, for the first time in a Tunisian context. We hypothesized that the farming system would significantly modulate the grain quality traits and immunogenic potential, and that this effect would be genotype-dependent, with ancient varieties potentially responding differently from modern ones.

## 2. Materials and Methods

### 2.1. Plant Material

Thirteen durum wheat varieties, representative of Tunisian populations cultivated throughout the 20th and 21st century, were used in this study. These varieties encompass a wide genetic diversity and include ancient varieties (Chili, Biskri, Mahmoudi, and Jenah Khotifa) and modern varieties (Karim, Razzak, Khiar, Maali, Salim, INRAT100, Dhahbi, Monastir, and Saragolla). The main characteristics of the wheat varieties studied are detailed in Table 1.

### 2.2. Experimental Site

The comparative evaluation of durum wheat varieties was conducted in field conditions during the 2023–2024 cropping season at the Gnadil experimental station in Beja (Tunisia). The site is characterized by a Mediterranean climate within the sub-humid bioclimatic zone, featuring cold winters and very hot, dry summers. Its geographical coordinates are 36°44′05″ N and 9°13′35″ E. During this study, the climatic conditions included 528 mm of cumulative rainfall, an average minimum temperature of 8.5 °C, and an average maximum temperature of 26.9 °C. The experiment followed a split-plot design with three replications. The main factor was the cropping system (organic vs. conventional), while the secondary factor was the durum wheat variety, randomized within each treatment. Each replication consisted of two subplots—one managed under conventional farming and the other under organic farming—containing elementary plots of 3 m × 1 m with six rows spaced 20 cm apart. The total experimental area was 234 m^2^. Soil analyses were performed on a clay-loam Vertisol (pH of 7.2) containing, on average, 48% clay, 30% loam, and 21% sand. The soil also contained 184 ppm total nitrogen, 3.92 ppm available phosphorus, 1.22% organic matter, and 17% CaCO_3_, and had an electrical conductivity of 0.92 mmhos/cm. Importantly, the field had not received synthetic chemical inputs for at least three years, complying with organic farming standards. After the harvest, seeds from each replicate were cleaned, homogenized, and ground to <500 μm using a laboratory mill. The resulting flours were stored at 4 °C until analysis.

### 2.3. Analysis of Total Protein Content and Protein Fractions

The protein content was determined according to AFNOR NF V03-050 [19].

Albumin, globulin, and gliadin fractions were sequentially extracted according to the procedure of Lookhart and Bean [20], with minor modifications. Briefly, 100 mg of flour were first extracted with 500 μL of deionized water for 30 min and centrifuged at 805 *g* for 5 min. The supernatant was collected, and the pellet was re-extracted twice with 400 μL of deionized water under the same conditions. The three supernatants were combined and considered as the albumin fraction.

The remaining pellet was then extracted with 400 μL of 0.5 M sodium chloride for 30 min and centrifuged at 805 *g* for 5 min. The supernatant was collected and the extraction was repeated twice. The three supernatants were pooled to obtain the globulin fraction. Subsequently, the pellet was extracted three times with 400 μL of 70% (*v*/*v*) aqueous ethanol for 30 min and centrifuged for 5 min at 805 *g*. The combined supernatants constituted the gliadin fraction.

Glutenins were extracted from the residual pellet as previously described [21]. Two successive extractions were performed, using 1 mL of a solution containing 50% (*v*/*v*) propan-1-ol, 2 M urea, 1% (*w*/*v*) dithiothreitol, and 0.05 M Tris–HCl (pH 7.5), followed by incubation at 60 °C for 60 min. The suspensions were centrifuged at 15,000 *g* for 20 min, and the two supernatants were collected and combined.

The extracted protein fractions were quantified using the Bradford method [22], with bovine serum albumin (BSA) (Sigma, Dorset, UK) as the standard.

All samples were analyzed in duplicate.

RP-HPLC-UV characterization of protein fractions was performed according to Li et al. [21], with minor modifications. Each fraction was analyzed using a JUPITER 5 μm C4, 300 Å, 250 × 2 mm RP column coupled to an HPLC-UV system with a dual-wavelength detector (Alliance 2695 model 2487, Waters, Milford, MA, USA). Separation was achieved using a linear gradient of phase A (0.1% *v*/*v* trifluoroacetic acid in water) and phase B (0.1% *v*/*v* trifluoroacetic acid in acetonitrile) from 100% A to 100% B over 0–40 min, followed by the washing and re-equilibration steps. The flow rate was 0.2 mL/min, the column temperature was maintained at 35 °C, and the injection volumes were 25 μL for albumin, gliadin, and glutenin, and 50 μL for globulin. UV detection was performed at 214 nm.

Quantification was performed using an external calibration curve with BSA. The peak assignment followed the previously reported criteria [21,23].

### 2.4. Determination of Total Amino Acid (AA) Composition

The AA profile was determined after acid hydrolysis. Samples (30 mg) were hydrolyzed using 6 M HCl at 110 °C for 24 h in sealed vials under an inert N_2_ atmosphere. After hydrolysis, HCl was removed using a Rotavapor (R-300, Büchi Labortechnik AG, St. Gallen, Switzerland). The residue was dissolved in 0.2 M lithium citrate buffer (pH 3.37) containing norleucine (250 nmol/mL) as the internal standard, centrifuged at 3500 *g* for 10 min, and filtered through a 0.22 μm filter.

For the determination of sulfur-containing AA, samples were subjected to performic acid oxidation prior to hydrolysis. A volume of 15 mL of performic acid solution (1 mL of 30% H_2_O_2_ and 9 mL of 98% formic acid) was added to the samples containing 30 mg of protein. The mixture was incubated at 4 °C for 18 h under agitation. Oxidation was stopped by adding 0.3 mL of 48% HBr per mL of performic acid while keeping the samples in an ice bath. Bromine was removed by vacuum evaporation using a Rotavapor, after which the samples were subjected to acid hydrolysis, as described above.

The separation and quantification of AA were performed using an automated amino acid analyzer (LKB 4400, LKB-Produkter AB, Stockholm, Sweden) equipped with a cation-exchange resin column (Ultropac 8, Pharmacia, Uppsala, Sweden). At the column outlet, amino acids reacted with ninhydrin, and the resulting colored complexes were detected by using a dual-channel photometer (440 nm for proline; 570 nm for the remaining amino acids) connected to a recorder and an integrator. Identification was based on retention times, and quantification was performed using peak areas established by the injection of a standard AA solution.

### 2.5. Determination of Gluten Content

Wet gluten content and gluten index were measured following the ISO 21415-2:2015 procedure [24], using an automatic Glutomatic device (Glutomatic 2200, Perten Instruments, Hägersten, Sweden). The fraction remaining on the sieve after determining the wet gluten content was centrifuged (6000 ± 5 rpm) and weighed to calculate the gluten index. The dry gluten content was obtained by the rapid drying of wet gluten, according to ISO 21415-3:2006 [25].

### 2.6. Determination of Starch Content

Starch was isolated from wheat flour by following the procedure of Chen et al. [26], with slight adjustments. Briefly, 100 g of wheat flour were mixed with an amount of water corresponding to 60% of its hydration capacity for 2 min to form a dough, which was then allowed to rest for 8 min. An additional 100 mL of water were incorporated, and the dough was stirred for 25 min. The mixture was transferred to a container with 500 mL of water and stirred for 35 min to promote gluten agglomeration. The resulting suspension, along with an additional 500 mL of water, was passed through vibrating sieves with decreasing mesh sizes (400, 250, 125, 90, and 50 µm). Gluten and fiber were retained on the sieves, while starch remained in the filtrate. The starch suspension was allowed to settle for 24 h at 4 °C, and the supernatant was decanted. The remaining suspension was centrifuged at 3000 *g* for 10 min, and the supernatant was discarded. The sediment consisted of a yellow-brown upper layer (sludge fraction) resting above a starch layer. The upper layer was removed and the starch fraction was re-suspended in water and subjected to a third centrifugation step. Approximately 50 g of purified starch were recovered and dried at 40 °C for 24 h.

The total starch (TS) and amylose content (ASE) were determined using the iodine colorimetric method [27].

### 2.7. Immunochemical Reactivity Against Anti-Gliadin Antibodies

#### 2.7.1. Production of Gluten Extracts

The gluten fraction was extracted from wheat flour (300 mg) by using 1.5 mL of 60% ethanol (*v*/*v*). The mixtures were vortexed, sonicated for 5 min in an ultrasonic water bath (J. P. Selecta, Barcelona, Spain), and incubated under agitation (1.000 rpm, 1 h, 20 °C) in a thermomixer (ThermoMixer, Eppendorf, Hamburg, Germany). The extracts were centrifuged (13,000 rpm, 5 min, 20 °C) in a 5424 R centrifuge (Eppendorf, Germany) and supernatants were taken. The protein content was measured in supernatants using Pierce^TM^ 660 reagent (ThermoFisher Scientific, Waltham, MA, USA). Extracts were stored at −20 °C until use.

#### 2.7.2. Sodium Dodecyl Sulfate Polyacrylamide Gel Electrophoresis (SDS-PAGE)

Gluten extracts were diluted in NuPAGE^®^ LDS sample buffer (1:3 *v*/*v*) and were loaded (15 µg protein/well) on NuPAGE^®^ Novex 4–12% Bis-Tris Gels (Thermo Fisher Scientific, Waltham, MA, USA). The pre-stained PageRuler^TM^ Plus (Thermo Fisher Scientific) was used as a protein marker. The gels were placed in an XCell-sure lock Mini-Cell (Thermo Fisher Scientific), run at 200 V for 35 min, stained with SimplyBlue SafeStain (ThermoFisher Scientific), and distained with distilled water.

#### 2.7.3. Western Blotting

After SDS-PAGE, the proteins were transferred onto a PVDF membrane using a Trans-Blot Turbo Transfer System (Biorad Laboratories, Hercules, CA, USA). PVDF membranes were blocked with 5% defatted dry milk (Nestlé España, Barcelona, Spain) for 1 h and washed three times (10 min) with Tris Buffer Saline-Tween 20 (TBST). Then, membranes were incubated overnight with a rabbit anti-gliadin antibody labeled with horseradish peroxidase (Sigma Aldrich, Madrid, Spain). After washing, PVDF membranes were incubated with horseradish peroxidase chemiluminescent substrate (Pearce ECL Western Blotting Substrate, Thermo Fisher Scientific, Waltham, MA, USA) for 5 min at room temperature. Pictures of membranes were taken using a ChemDoc XRS+ Imaging System (Bio-Rad, Madrid, Spain).

### 2.8. Peptidomic Analysis

Intestinal digests were obtained by applying the standardized static INFOGEST 2.0 in vitro gastrointestinal digestion protocol [28] to the selected durum wheat samples (INRAT 100 and Chili), as previously described. The resulting small-intestinal phase digests were immediately processed for LC–MS/MS peptidomic analysis. The samples were resuspended in 1 mL of 0.1% trifluoroacetic acid, vortexed for 10 s, and sonicated for 3 min, and the residual solid material was mechanically disrupted using ultrafine pipette tips. The procedure was repeated, and the samples were finally centrifuged at 13,000× *g* at room temperature for 2–3 min. Aliquots of 100 µL of the supernatants were collected, and the remaining material was retained. The collected aliquots were purified using OMIX C18 reversed-phase columns (Agilent Technologies, Madrid, Spain). After drying, the samples were resuspended in 12 µL of 0.1% formic acid and the peptide concentration was determined using a Qubit fluorometer. Approximately 1 µg of peptide was injected into a Q-Exactive mass spectrometer (Thermo Scientific, Waltham, MA, USA) in all cases.

Peptides were first trapped on an Acclaim PepMap 100 precolumn (Thermo Scientific) and subsequently separated on an Acclaim PepMap 100 C18 analytical column (50 cm length, 75 µm internal diameter, 2 µm particle size; Thermo Scientific). Peptide separation was performed by using a 136 min linear gradient, as follows: 5 min at 2% buffer B; 100 min from 2% to 20% buffer B; 20 min from 20% to 32% buffer B; 1 min from 32% to 95% buffer B; and 10 min at 95% buffer B. Buffer A consisted of 0.1% formic acid in water, and buffer B consisted of 0.1% formic acid in 80% acetonitrile. The flow rate was set at 300 nL/min, using a nano Easy-nLC 1000 system (Proxeon) coupled to a nanoelectrospray ion source (Thermo Scientific).

Mass spectra were acquired on a Q-Exactive mass spectrometer (Thermo Scientific) operating in positive ion mode. Full-scan mass spectra were acquired over an *m*/*z* range of 375–1500 at a resolution of 70,000 (at *m*/*z* 200) in the Orbitrap analyzer. The ten most intense precursor ions from each full scan were selected for fragmentation by higher-energy collisional dissociation (HCD). Fragment ion spectra were acquired at a resolution of 17,500 (at *m*/*z* 200). Singly charged ions and ions with unassigned charge states were excluded from fragmentation. Dynamic exclusion was applied with a duration of 20 s.

Raw spectral files (*.raw) were searched against the UniProt *Triticum* reviewed database (512 sequences) and the SwissProt *Viridiplantae* database (41,948 sequences). Database searches were performed using Proteome Discoverer software (version 3.0.0.757, Thermo Scientific), with the Mascot search engine. No specific protease was selected, and no fixed amino acid modifications were defined.

Precursor and fragment ion mass tolerances were set at 10 ppm and 0.02 Da, respectively. Peptide identifications were validated using the Percolator algorithm with a q-value threshold of ≤0.01.

### 2.9. Mapping of Immunogenic Epitopes Against Identified Peptides

Immunogenic epitope annotation was performed following the workflow described by Lavoignat et al. [29]. Briefly, all experimentally validated allergenic and celiac-disease-related epitopes were exported from the Immune Epitope Database (IEDB) and compiled into a reference epitope library. The epitope dataset included linear peptide sequences associated with confirmed immunogenic activity.

The identified peptide sequences obtained from LC–MS/MS analysis of intestinal digests were mapped against the epitope library, using exact sequence matching to identify peptides containing complete epitope sequences. For each peptide, the presence or absence of at least one complete epitope was recorded. Peptides containing partial epitope matches were not considered positive.

For each sample, the total number of identified peptides and the number of peptides containing at least one complete epitope were calculated. Results were expressed as the absolute number and percentage of epitope-containing peptides per intestinal digest. In addition, the number of peptide-spectrum matches (#PSMs) was used as a semi-quantitative proxy of peptide abundance. For each sample, cumulative PSMs associated with epitope-containing peptides and total PSMs were calculated. The relative abundance of immunogenic peptides was expressed as the percentage of PSMs attributed to epitope-positive peptides. This analysis was used as a complementary indicator to support qualitative comparisons among samples.

### 2.10. Statistical Analysis

Statistical analyses were performed using the SPSS software, version 27 (IBM Corp., Armonk, NY, USA). A two-way analysis of variance (ANOVA) was conducted using the GLM procedure to assess the effect of the farming system (two levels: organic vs. conventional) and variety (*n* = 13), and their interaction. Following this analysis, the means were compared by using Tukey’s post hoc test.

## 3. Results and Discussion

### 3.1. Total Protein Content

The protein content of the durum wheat varieties under organic and conventional farming is summarized in Table 2. The protein levels ranged from 12.40 to 15.50 g/100 g dm across the varieties studied. These values are consistent with those reported for Tunisian durum wheat genotypes under different nitrogen treatments (12.45–14.80 g/100 g dm) [30], for Polish wheat varieties cultivated under intensive or integrated technologies (13–15 g/100 g dm) [31] and for various Indian wheat varieties (9.32–12.60 g/100 g dm) [32].

The effect of the farming system varied depending on the variety. Organic farming resulted in lower protein levels than conventional farming in Karim, Razzak, Salim, Dhanhbi, and Monastir. The opposite trend was observed in Chili, Biskri, and Mahmoudi. For INRAT 100 and Saragolla, no significant differences in the protein content were detected between the two farming systems.

Protein is an indirect indicator of flour strength and bread-making potential [33]. However, it is a low-heritability trait that is strongly influenced by environmental conditions and agronomic practices, particularly nitrogen availability [34]. The present results indicate that the effect of the farming system on the durum wheat protein content was varietal-dependent, in agreement with previous studies. España-Fariñas et al. [35] reported a higher protein content under organic farming compared to conventional management in the Caaveiro wheat variety, whereas no such trend was observed in the Callobre variety. Similarly, no differences in protein content were detected in the Caaveiro variety grown under organic and conventional conditions in another study [36]. In contrast, several authors have reported a lower protein content in organically produced wheat flour compared with the conventionally produced ones [34,37]. This reduction has been attributed to decreased nitrogen availability under organic management, which may induce metabolic shifts toward carbon-containing compounds and nitrogen-based secondary metabolites [38]. Such mechanisms may explain the lower protein content observed in the organically cultivated Karim, Razzak, Salim, Dhahbi, and Monastir varieties compared with their conventionally grown counterparts. Conversely, Chili, Biskri, and Mahmoudi appear to exhibit a greater capacity for nitrogen remobilization under limited soil nitrogen conditions, as well as a higher nitrogen uptake efficiency, which could account for their protein levels under organic farming. The similar protein content of INRAT 100 and Saragolla under both farming systems suggest that, in these varieties, genetic factors play a more important role than agronomic practices in determining the protein content. Le Gouis [39] proposed the use of physiological markers as field diagnostic tools to guide nitrogen fertilization according to variety groups oriented toward nitrogen-efficient production. This approach appears particularly relevant, given the substantial variability in varietal responses with respect to protein content observed in the present study.

According to Cabas-Lühmann et al. [40], durum wheat is considered to have good technological and culinary quality when grain protein content is ≥13%. Most of the varieties evaluated in this study under both conventional and organic farming systems exceeded this threshold, indicating their suitability for producing high-quality foods such as pasta, for which the protein content is a critical determinant of the final product quality.

### 3.2. Protein Composition

The technological quality of wheat grains is closely associated with their storage protein composition [41]. In this context, this study evaluated the effect of the farming system on the protein composition of different wheat varieties.

The content of the protein fractions extracted from the different wheat varieties is presented in Table 3. As expected, gluten proteins (gliadins and glutenins) constituted the predominant fractions. Among them, γ-gliadin and low-molecular-weight (LMW) glutenins were the most abundant, ranging from 43.48 to 84.36 and from 31.16 to 63.75 µg/mg, respectively. Albumins (27.32–28.74 µg/mg) and globulins (23.61–26.04 µg/mg) were present at comparable levels, whereas ω-gliadins represented the minor fraction (1.80–3.72 µg/mg). These values are consistent with those that were previously reported for landraces and modern genotypes [6], as well as for spring wheat grown under regulated deficit irrigation [42]. It should be noted that a Bradford assay can exhibit variable responses to different protein types, which represents a potential limitation of the quantification of protein fractions.

No significant differences (*p* > 0.05) were observed in the albumin and globulin contents between the conventional and organic systems, with the exception of Biskri, which exhibited significantly higher globulin levels under organic conditions (*p* ≤ 0.05). These results support earlier findings indicating that genotype exerts a stronger influence than the farming system on these fractions [43,44].

In contrast, gluten protein fractions were more sensitive to farming practices, although the direction and magnitude of these effects were variety-dependent. For gliadins, Karim and Salim showed higher αβ- and γ-gliadin levels under conventional farming, whereas Mahmoudi accumulated greater amounts of these fractions under organic conditions. Razzak and Dhahbi displayed higher γ-gliadin levels when grown conventionally, but both varieties, together with Monastir, showed increased αβ-gliadins under organic management. Maali and Jenah Khotifa also accumulated more γ-gliadins under organic conditions. Conversely, INRAT 100, Chili and Biskri presented higher ω- and γ-gliadin contents when cultivated organically. These heterogeneous responses contrast with the consistent increases in all protein fractions under organic farming that have been reported in Indian wheat varieties [45], highlighting that the impact of the farming system on the gluten proteins depends largely on the genotype. Similarly, although higher nitrogen availability—typically associated with conventional farming—has been shown to increase the total wheat protein content, its impact on the gliadin and glutenin fractions appears to be dependent on the cultivar [46]. The variability observed in the present study reinforces the predominant role of genetic factors (Table A1, Appendix A) in regulating the gliadin and glutenin accumulation observed in wheat in previous studies [44,47]. Since gliadins contribute to dough extensibility and stickiness and glutenins to strength, stability and resistance [32], identifying farming conditions that enhance both fractions may ultimately improve the technological performance of wheat in breadmaking and pasta production.

### 3.3. Quality Traits Related to Gluten

Table 4 summarized the content of wet and dry gluten, as well as the gluten index of all wheat varieties studied. The wet gluten content spanned from 28.21% to 34.97%. All varieties showed higher wet gluten levels under conventional farming, with the exception of the Chili, Biskri, Mahmoudi and Jenah Khotifa varieties, which displayed an opposite pattern, with significantly (*p* ≤ 0.05) higher wet gluten content under organic management.

Regarding dry gluten, it ranged from 8.9% to 19.1%, with the Maali, Salim, INRAT100, Dhahbi, Monastir, and Saragolla varieties showing greater values under conventional farming, while Karim, Razzak, Chili, Biskri and Jenah Khotifa exhibited a higher dry gluten content under organic farming. In contrast, no differences between both farming systems were observed for Khiar and Mahmoudi.

On the other hand, the gluten index ranged from 45.03% to 86.00%, and it was affected by the farming system in different ways, depending on the variety: Karim, Razzak, Khiar, Maali, Salim, Dhahbi, and Monastir showed higher values of this parameter under conventional management, whereas Chili, Jenah Khotifa, and Mahmoudi exhibited a higher gluten index under organic farming. Conversely, no significant differences in the gluten index were observed between the two farming systems for Saragolla.

The results regarding wet gluten, dry gluten, and the gluten index align with those previously reported for different wheat varieties [48,49,50,51], and reinforce the recent findings highlighting that nitrogen availability and management quantitatively modulate storage protein synthesis [52]. The results of this study revealed that the effect of the farming system on quality traits related to gluten strongly depends on the variety considered, confirming the important influence of the genetic factor, as previously reported [48]. Chili, Biskri, and Jenah Khotifa showed higher contents of wet and dry gluten, as well as a higher gluten index under organic farming, suggesting a better adaptation of the old wheat varieties to low-input farming systems compared to the modern ones.

Gluten represents the main storage protein fraction of wheat, and plays a central role in conferring the unique technological properties that make wheat particularly suitable for the production of leavened products [53]. Wet gluten is an indicator for dough characteristics, and differences in this parameter affect the properties of flour-based products [54]. An elevated wet gluten content is typically associated with a higher water absorption capacity. The greater the water retained by gluten, the larger the difference between the wet and dry gluten, reflecting superior gluten quality. This parameter constitutes a critical techno-functional indicator for evaluating the gluten performance in breadmaking and other cereal-based products [55]. The gluten index measures the gluten quantity and quality and is related to the elasticity grade and extensibility of flour [56]. It has been reported that for baking purposes, the wet gluten content and gluten index in wheat grains should not be lower than 27% and 60%, respectively [49]. All wheat varieties studied in the present work had a wet gluten content above this threshold and Karim, Razzak, INRAT 100, Dhahbi, Saragolla, Chili, Biskri, Mahmoudi, and Jenah Khotifa showed gluten index values higher than 60%, indicating their excellent technological potential to be included in baking products.

### 3.4. Amino Acid Composition

The amino acid composition of wheat varieties cultivated under conventional and organic systems is presented in Table 5 and Table 6.

The total essential amino acid content (Table 5) ranged from 3.00 to 5.05 g/100 g dm. Leucine was the most abundant essential amino acid in all varieties (0.72–0.98 g/100 g dm), followed by valine (0.47–0.80 g/100 g dm) and phenylalanine (0.46–0.76 g/100 g dm), irrespective of the cropping system (Table 5). In contrast, methionine (0.11–0.26 g/100 g dm) and lysine (0.29–0.70 g/100 g dm) were present in the lowest amounts, in agreement with previous studies [31].

The farming system exerted a noticeable effect on the essential amino acid content. The varieties Karim, Razzak, Khiar, Maali, Salim, Dhahbi, and Monastir showed higher total essential amino acid levels under conventional farming, whereas INRAT 100, Chili, Biskri, Mahmoudi, and Jenah Khotifa exhibited higher contents under organic farming. No significant differences (*p* > 0.05) were observed between the two farming systems for the Saragolla variety.

Regarding non-essential amino acids (Table 6), the total contents varied from 7.84 to 12.20 g/100 g dm. A similar pattern to that observed for essential amino acids was detected, with most varieties showing higher contents under conventional farming. However, the ancient varieties, Chili, Biskri, Mahmoudi, and Jenah Khotifa, as well as INRAT 100, exhibited higher levels under organic farming. Glutamic acid was the predominant non-essential amino acid (3.95–6.22 g/100 g dm), followed by proline (0.95–1.55 g/100 g dm), in agreement with previous reports [2,31,32,50].

Overall, the essential and non-essential amino acid contents observed in the studied wheat varieties were consistent with those reported for Polish spring wheat grains grown under both conventional and organic farming systems [2].

Previous studies have suggested that organic farming may increase the lysine, valine, tryptophan, and methionine contents compared to conventional systems [2,57]. Conversely, organic farming has also been associated with a significant reduction in several amino acids, including threonine, serin, glutamic acid, alanine, leucine, tyrosine, phenylalanine, histidine, and arginine [55]. In the present study, ancient wheat varieties grown under organic farming generally exhibited higher levels of most individual amino acids, whereas modern genotypes showed similar or lower amino acid contents under organic conditions compared to conventional farming. These findings highlight the strong influence of the interaction between genotype and agronomic practices on individual amino acid profiles (Table A2, Appendix A).

Nitrogen fertilization has been reported to significantly increase the protein and the levels of both essential and non-essential amino acids in wheat [58]. Consequently, higher amino acid contents might be expected under organic farming systems. However, cultivar-specific responses to nitrogen availability have been documented [58,59], which may explain the contrasting behavior observed between modern and ancient wheat varieties in response to farming systems.

Amino acids play a fundamental role in determining the quality of cereal products by influencing both their nutritional value and their metabolic properties [60]. They serve as key substrates for dough microorganisms and contribute notably to the flavor of wheat-based products [61]. In this context, the cultivation of ancient durum wheat varieties under organic farming conditions appears to be a promising strategy to enhance the nutritional quality of this cereal and, consequently, of the food products derived from it.

### 3.5. Starch, Amylose, and Amylopectin Contents

Table 7 shows the total starch, amylose and amylopectin contents in the different wheat genotypes grown under both conventional and organic farming systems. The starch content varied from 46.92 to 87.53 g/100 g, values comparable to those previously reported for different wheat varieties [34,52]. The production systems notably affected the starch levels. Compared with the conventional system, organic farming resulted in a significant reduction (*p* ≤ 0.05) in the starch content for all the varieties, except Khiar and Jenah Khotifa, for which no significant differences (*p* > 0.05) were observed between both cropping systems. In contrast, other studies have reported higher starch contents in organically grown wheat compared with conventionally produced wheat [34,52]. An inverse relationship between the protein and starch contents in cereals has been reported [34,62]. In general, higher nitrogen availability promotes protein synthesis, while nitrogen limitation favors the accumulation of reserve carbohydrates such as starch [63]. These trends are consistent with the results observed for the ancient wheat varieties studied (Chili, Biskri, and Mahmoudi), in which organic farming resulted in higher protein and lower starch contents. However, in the remaining varieties, the reduced starch content under organic farming was not accompanied by an increase in the protein content, suggesting that additional factors, including genotype and environmental growing conditions, may influence the starch synthesis in wheat, as previously reported.

Starch is the primary reserve carbohydrate in wheat and the major determinant of the quality of wheat-based products [64]. Wheat varieties with higher starch contents are often associated with an increased yield, which is likely due to enhanced carbohydrate metabolism and more efficient translocation of photo-assimilates to the grain [62]. In this context, the results of the present study suggest that modern wheat varieties cultivated under conventional farming systems may be more suitable for certain industrial applications.

Amylose and amylopectin contents ranged from 16.03 to 46.99 g/100 g and 53.01 to 83.97 g/100 g, respectively (Table 7), in agreement with other studies [65]. The results of the present work revealed a significant interaction between the cultivation system and genotype affecting amylose and amylopectin content (Table A3, Appendix A). Most wheat varieties showed higher amylose content under organic farming, with the exception of the variety Salim, where conventional farming led to higher content, and Monastir, where no differences between the two farming systems were found. Reduced nitrogen, typically found in organic cultivation, limits protein synthesis and alters the photoassimilate allocation, favoring the formation of linear starch chains (amylose) over branched chains (amylopectin). Additionally, nutrient and environmental stresses in organic systems could modulate starch-branching enzyme activity, further increasing amylose proportion [66]. More variability was observed in the amylopectin content, where most of the varieties exhibited a higher content under conventional farming, whereas only the variety Monastir showed higher amylopectin levels under organic farming. The Karim, Khiar, Salim, Biskri and Jenah Khotifa varieties presented similar amylopectin levels under both farming systems. Multiple studies have reported that variety identity exerts a stronger influence than the farming system or environmental conditions on cereal starch composition. Li and Liu [67] demonstrated that variety significantly affects amylose and amylopectin chain length distribution. Similarly, Nhan and Copeland [68] confirmed that wheat genotype is the primary determinant of starch physicochemical properties, surpassing environmental influences.

A high amylose-to-amylopectin ratio generates a higher fraction of resistant starch and it is related to potential health benefits due to slower starch digestion and metabolic benefits, such as prolonged satiety and improved glucose regulation [69]. Moreover, a high amylose-to-amylopectin ratio content increases the water-binding capacity of flours and improves the elasticity and resistance to deformation of doughs [70,71]. The ancestral varieties grown under organic farming systems appear to be promising candidates for food industry applications, owing to their higher amylose and lower amylopectin contents.

### 3.6. Protein Profile and Immunoreactivity of Wheat Varieties

**Figure 1:**
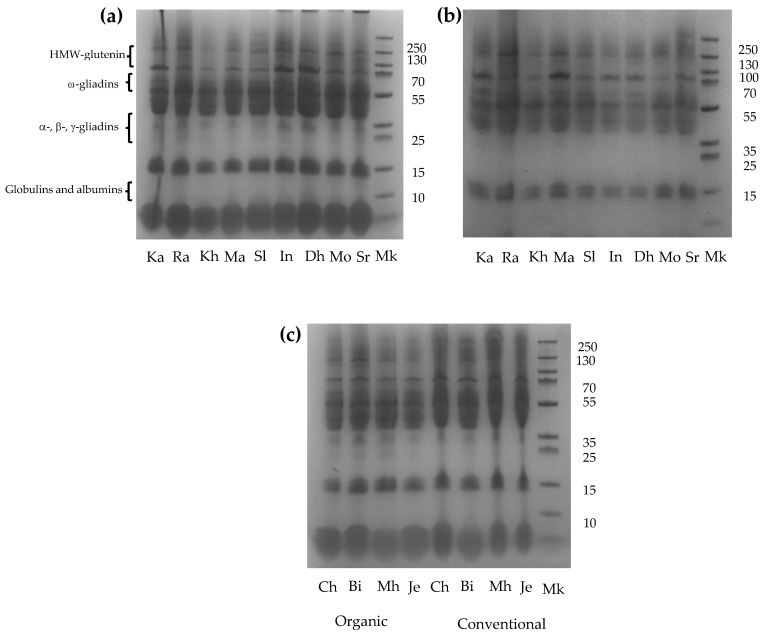
Protein profile of whole semolina of wheat varieties grown under conventional and organic farming systems. (**a**) Modern varieties grown under organic system; (**b**) modern varieties grown under conventional system; and (**c**) ancestral varieties grown under organic and conventional system. Ka: Karim, Ra: Razzak, Kh: Khiar, Ma: Maali, Sl: Salim, In: INRAT 100, Dh: Dhahbi, Mo: Monastir, Sr: Saragolla, Ch: Chili, Bi: Biskri, Mh: Mahmoudi, and Je: Jenah Khotifa. Mk: molecular weight marker.

Figure 1 illustrates the gluten protein profiles of the analyzed wheat varieties. Bands corresponding to polypeptides with molecular weights between 16 and 130 kDa were detected. These bands were tentatively assigned to HMW glutenin subunits and their aggregates (70–126 kDa); ω-gliadins (53–70 kDa); and α-, β-, and γ-gliadins/LMW glutenins (35–55 kDa). Bands below 15 kDa were attributed to the globulins and albumins extracted under applied conditions. All varieties exhibited electrophoretic patterns that were consistent with those previously reported [32,72,73].

Overall, the electrophoretic profiles were qualitatively similar among the analyzed varieties; however, differences in band intensities revealed quantitative variations in specific proteins. The Khiar, Maali, Monastir, and Salim varieties showed lower levels of HMW glutenin subunits under both farming systems compared with the other varieties (Figure 1a,b).

Regarding farming systems, the Khiar, Razzak, Khiar, Maali, Salim, INRAT 100, Dhabi, Monastir, and Saragolla varieties, grown under organic farming, exhibited higher overall gluten protein content than their conventionally grown counterparts, as indicated by increased band intensities (Figure 1a,b). In contrast, no apparent differences in the gluten protein content between farming systems were observed for the ancestral varieties of Chili, Biskri, Mahmoudi and Jenah Khotifa (Figure 1c). Similar variations in the relative proportion of gluten protein subfractions among wheat varieties have previously been reported [32,74] and may be attributed to genetic variability and environmental growing conditions.

**Figure 2:**
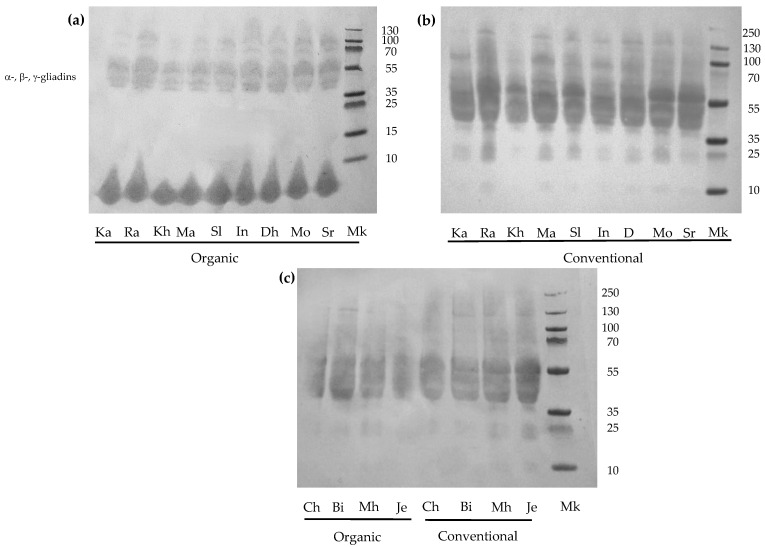
Immunochemical reactivity against anti-gliadin antibodies of whole semolina of wheat varieties grown under conventional and organic farming systems. (**a**) Modern varieties grown under organic system; (**b**) modern varieties grown under conventional system; and (**c**) ancestral varieties grown under organic and conventional system. Ka: Karim, Ra: Razzak, Kh: Khiar, Ma: Maali, Sl: Salim, In: INRAT 100, Dh: Dhahbi, Mo: Monastir, Sr: Saragolla, Ch: Chili, Bi: Biskri, Mh: Mahmoudi, and Je: Jenah Khotifa. Mk: molecular weight marker.

Figure 2 shows the immunochemical reactivity of gluten extracts against anti-gliadin antibodies. Strong immunoreactive bands corresponding to α-, β and γ-gliadins (35–55 kDa) were observed in all varieties, whereas weaker reactivity was detected for ω-gliadins (53–70 kDa). Although the polyclonal antibody was expected to recognize the different gliadin fractions (α-, β-, γ-, and ω-), similarly, it exhibited varying reactivity across these fractions.

Wheat varieties cultivated under organic farming conditions showed apparently lower immunoreactivity against anti-gliadin antibodies compared with those grown under conventional farming. In agreement with these findings, previous studies have shown that cultivation systems can influence the allergenic potential of wheat. In particular, organic farming has been associated with an increase in the QQQPP peptide, one of the main IgE-finding epitopes in individuals with wheat hypersensitivity, and a reduction in the panallergenic profilin Tri a 12 [16]. Reduced nitrogen availability under organic farming may modulate the expression of certain gliadin genes, resulting in a decreased accumulation of highly immunogenic epitopes. Additionally, wheat breeding strategies, nitrogen-based fertilization, and the use of certain pesticides have been reported to enhance wheat immunogenicity [75], which may contribute to the differences observed between the conventional and organic systems.

According to our results, no marked differences in immunoreactivity were observed between the modern and old wheat varieties. Similarly, Boukid et al. [76] reported high variability in the content of celiac-related immunogenic and toxic peptides among wheat varieties, reflecting substantial genetic diversity, but did not find evidence of higher immunogenicity in modern varieties compared with the older ones. Lavoignat et al. [29] also described genotype-dependent differences in celiac-disease-related epitopes after in vitro digestion, highlighting the significant influence of the genotype on wheat’s immunogenic potential. It should be noted that, in the present study, immunochemical reactivity was assessed by Western blotting using anti-gliadin antibodies, which may explain the discrepancies with studies based on ELISA assays or LC-MS quantification of immunotoxic peptides.

It should be noted that the present analysis was strictly qualitative and was conducted as an initial approach preceding the peptidomic analysis. To further investigate the immunogenic potential of the wheat varieties studied, one modern (INRAT 100) and one ancestral (Chili) durum wheat variety grown under both conventional and organic farming systems were subjected to in vitro digestion, and their resulting peptidomic profiles were subsequently analyzed.

### 3.7. Peptidomic Profile: UHPLC/ESI-MS/MS Analysis

The *in vitro* peptidomic analysis revealed clear differences in both the occurrence and relative abundance of immunogenic peptides across wheat varieties and farming systems (Supplementary Materials). For the Chili variety, the conventionally grown sample (Chili C) contained 139 immunogenic peptides out of 1665 identified peptides, corresponding to 8.35% immunogenic peptides (Table 8). When weighted by peptide-spectrum matches (PSMs), these peptides accounted for 17.41% of the total peptide abundance (2841 immunogenic PSMs out of 16,317 total PSMs). In contrast, the same genotype cultivated under organic conditions exhibited 47 immunogenic peptides among 998 identified peptides (4.71%), representing 4.68% immunogenic abundance (192 immunogenic PSMs out of 4101 total PSMs). For the INRAT 100 variety, the conventionally cultivated sample (INRAT100 C) displayed 57 immunogenic peptides among 851 identified peptides (6.70%), corresponding to 7.57% immunogenic abundance (298 immunogenic PSMs out of 3939 total PSMs). Under organic farming (INRAT100 O), 33 immunogenic peptides were detected among 622 total peptides (5.31%), accounting for 5.56% of immunogenic abundance (175 immunogenic PSMs out of 3145 total PSMs). Together, these results indicate that the farming system influences both the richness (number of peptides containing at least one immunogenic epitope) and the relative abundance of immunogenic peptides, with a particularly pronounced effect observed for the Chili variety. More broadly, the data demonstrate that both the genotype and agronomic conditions modulate the peptide signature released after simulated gastrointestinal digestion.

The higher proportion and abundance of immunogenic peptides observed in conventionally cultivated samples compared with their organic counterparts are consistent with recent findings by Lavoignat et al. [29], who reported genotype × environment interactions affecting the protein digestibility and the release of epitopes associated with celiac disease and wheat allergenicity. Those authors showed that farming conditions can alter peptide release profiles during digestion, although no single peptide signature clearly discriminates genotypes with high or low digestibility. Our observations align with this concept: Chili and INRAT100 exhibit distinct peptide profiles, and the farming system amplifies these differences. Environmental factors such as fertilization regime, water availability, and abiotic stress are known to influence the composition and proportion of storage proteins, particularly gliadins and glutenins, as shown in Table 3, thereby affecting the generation of immunogenic peptides during digestion. For example, Ronga et al. [77] demonstrated that agronomic conditions directly modulate the content of toxic and immunogenic gluten peptides in wheat grain. Importantly, immunogenic abundance expressed as a PSM-weighted percentage does not merely reflect the number of immunogenic peptides detected, but also their relative dominance within the peptide pool. In some cases, a limited number of peptides may account for a disproportionately large fraction of the total peptide signal, potentially increasing its immunological relevance [78,79]. Although PSMs provide only a semi-quantitative approximation of abundance, this metric enables meaningful comparative assessment across samples in the absence of intensity-based proteomics data. Absolute quantification using labeled standards could provide additional confirmation of these findings.

Finally, the detection of peptides containing known immunogenic epitopes does not necessarily imply pathogenicity *in vivo*. Functional immunological validation, such as T-cell activation assays or clinical evaluation, remains essential to establish a causal relationship between peptide occurrence and immune responses in sensitive individuals and patients with celiac disease [13].

## 4. Conclusions

This study shows that the effects of a farming system on durum wheat grain quality and immunogenic potential are variety-dependent. Organic farming did not induce consistent changes in protein content or gluten quality across genotypes, but was generally associated with reduced starch accumulation. Organically grown wheat exhibited lower immunochemical reactivity against anti-gliadin antibodies. Peptidomic analysis confirmed a reduced number and relative abundance of celiac-disease-related immunogenic peptides in organically cultivated samples compared with conventionally grown counterparts, with stronger effects in traditional varieties. These results highlight a significant genotype × farming system interaction in shaping protein composition and peptide release during digestion. Overall, combining appropriate varietal selection, particularly traditional durum wheat varieties, with organic management may enhance the grain quality while lowering the immunogenic peptide content, supporting sustainable, health-oriented wheat production. Future work should focus on validating the reduced immunogenic potential of these variety-farming system combinations by using T-cell activation assays. 

## Figures and Tables

**Table 1:** Characteristics of the wheat varieties included in the study.

Varieties	Origin	Registration Date	Main Characteristics
Chili	France	1932	Medium performance (15–20 q/ha), resistance to septoria and yellow rust
Biskri	Algeria	1909	Medium performance (15–20 q/ha), drought tolerance and very high semolina quality
Mahmoudi	Tunisia	1893	Medium performance (15–20 q/ha), drought tolerance and very high semolina quality
Jenah Khotifa	Tunisia	1915	Medium performance (15–20 q/ha), resistance to septoria and yellow rust
Karim	INRAT/CIMMYT	1980	Fairly resistant to septoria and brown rust
Razzak	INRAT-Tunisia	1987	Very productive, resistant to powdery mildew and fairly resistant to septoria and brown rust
Khiar	INRAT/CIMMYT	1992	Productive and relatively susceptible to brown rust and septoria diseases
Maali	INRAT-Tunisia	2007	Resistant to powdery mildew and fairly resistant to septoria and brown rust
Salim	INRAT-Tunisia	2009	High yield, resistance to septoria, yellow rust, and brown rust
INRAT100	CIMMYT/Tunisia	2017	Productive, resistance to septoria, yellow and brown rust, and drought tolerance
Dhahbi	INRAT-Tunisia	2018	Productive, resistance to powdery mildew and yellow rust and very high semolina quality
Monastir	SOSEM	2012	Productive, resistance to septoria and brown rust, and tolerant to drought
Saragolla	Italy	2004	High yield, resistance to septoria and rust

CIMMYT: International Maize and Wheat Improvement Centre; ha: hectare; INRAT: National Institute of Agricultural Research of Tunisia; q: quintals; and SOSEM: Selected Seeds Company.

**Table 2:** Protein content (g/100 g dm) in whole semolina of wheat varieties grown under conventional and organic farming systems.

Wheat Variety	Farming System	
	Conventional	Organic
Karim	15.50 ± 0.01 ^b^	13.42 ± 0.02 ^a^
Razzak	15.24 ± 0.04 ^b^	13.20 ± 0.05 ^a^
Khiar	12.47 ± 0.02 ^a^	12.22 ± 0.07 ^a^
Maali	12.66 ± 0.05 ^a^	12.31 ± 0.01 ^a^
Salim	13.31 ± 0.04 ^b^	12.75 ± 0.07 ^a^
INRAT100	14.36 ± 0.05 ^a^	14.38 ± 0.02 ^a^
Dhahbi	14.75 ± 0.06 ^b^	13.59 ± 0.00 ^a^
Monastir	13.80 ± 0.03 ^b^	12.82 ± 0.02 ^a^
Saragolla	14.03 ± 0.03 ^a^	14.02 ± 0.02 ^a^
Chili	12.40 ± 0.00 ^a^	13.38 ± 0.03 ^b^
Biskri	13.00 ± 0.02 ^a^	14.09 ± 0.04 ^b^
Mahmoudi	13.24 ± 0.02 ^a^	14.40 ± 0.04 ^b^
Jenah Khotifa	13.04 ± 0.03 ^a^	13.68 ± 0.01 ^a^

Values are expressed as mean ± standard deviation (*n* = 3). Different letters within the same row for a given variety indicate statistically significant differences (*p* ≤ 0.05, Tukey’s test).

**Table 3:** Protein fractions (µg/mg dm) in whole semolina of wheat varieties grown under conventional and organic farming systems.

Wheat Variety	Farming System	Albumins	Globulins	Gliadins			Glutenins	
				ω	αβ	γ	HMW	LMW
Karim	Conventional Organic	28.25 ± 0.26 ^a^ 28.28 ± 0.20 ^a^	23.63 ± 0.86 ^a^ 24.36 ± 0.94 ^a^	2.19 ± 0.04 ^a^ 2.25 ± 0.04 ^a^	13.60 ± 0.15 ^b^ 10.23 ± 1.01 ^a^	68.15 ± 0.21 ^b^ 50.50 ± 4.98 ^a^	34.15 ± 0.20 ^a^ 33.94 ± 0.26 ^a^	63.24 ± 0.26 ^a^ 63.75 ± 0.11 ^a^
Razzak	Conventional Organic	28.26 ± 0.64 ^a^ 27.64 ± 0.47 ^a^	24.50 ± 0.77 ^a^ 24.50 ± 0.91 ^a^	1.90 ± 0.04 ^a^ 1.90 ± 0.02 ^a^	8.98 ± 0.04 ^a^ 10.99 ± 0.96 ^b^	84.07 ± 0.11 ^b^ 48.79 ± 0.04 ^a^	32.53 ± 1.28 ^a^ 31.91 ± 0.17 ^a^	38.54 ± 2.48 ^b^ 31.16 ± 0.11 ^a^
Khiar	Conventional Organic	28.44 ± 0.66 ^a^ 28.02 ± 0.61 ^a^	24.07 ± 0.70 ^a^ 24.52 ± 0.37 ^a^	3.71 ± 0.06 ^a^ 3.72 ± 0.03 ^a^	6.53 ± 0.08 ^a^ 6.54 ± 0.03 ^a^	35.54 ± 0.06 ^a^ 43.48 ± 0.04 ^a^	31.40 ± 0.15 ^a^ 31.16 ± 0.11 ^a^	53.96 ± 1.48 ^b^ 48.51 ± 0.20 ^a^
Maali	Conventional Organic	27.96 ± 0.36 ^a^ 27.81 ± 0.77 ^a^	24.25 ± 0.42 ^a^ 23.80 ± 0.42 ^a^	1.80 ± 0.02 ^a^ 1.82 ± 0.03 ^a^	6.01 ± 0.04 ^a^ 6.03 ± 0.02 ^a^	45.11 ± 0.09 ^a^ 80.94 ± 6.84 ^b^	30.64 ± 0.10 ^a^ 30.73 ± 0.16 ^a^	54.00 ± 0.11 ^a^ 54.05 ± 0.08 ^a^
Salim	Conventional Organic	27.32 ± 0.75 ^a^ 27.95 ± 0.43 ^a^	24.26 ± 0.64 ^a^ 23.67 ± 0.62 ^a^	2.38 ± 0.04 ^a^ 2.39 ± 0.02 ^a^	5.71 ± 0.56 ^b^ 4.06 ± 0.57 ^a^	65.03 ± 0.08 ^b^ 46.15 ± 0.04 ^a^	31.82 ± 0.11 ^a^ 31.80 ± 0.14 ^a^	56.32 ± 0.13 ^a^ 56.37 ± 0.14 ^a^
INRAT 100	Conventional Organic	27.77 ± 0.43 ^a^ 28.28 ± 0.66 ^a^	23.61 ± 0.49 ^a^ 23.79 ± 0.65 ^a^	2.80 ± 0.03 ^a^ 3.50 ± 0.53 ^b^	9.17 ± 0.03 ^a^ 9.20 ± 0.03 ^a^	41.48 ± 0.07 ^a^ 53.42 ± 1.94 ^b^	33.24 ± 0.10 ^a^ 33.16 ± 0.15 ^a^	56.59 ± 0.18 ^a^ 56.60 ± 0.11 ^a^
Dhahbi	Conventional Organic	27.86 ± 0.97 ^a^ 28.25 ± 0.41 ^a^	24.31 ± 0.13 ^a^ 24.28 ± 0.94 ^a^	2.50 ± 0.03 ^a^ 2.49 ± 0.04 ^a^	7.78 ± 0.03 ^a^ 12.12 ± 0.5 ^b^	78.96 ± 2.28 ^b^ 60.31 ± 0.12 ^a^	35.13 ± 0.98 ^b^ 32.14 ± 0.07 ^a^	51.66 ± 0.14 ^a^ 51.66 ± 0.08 ^a^
Monastir	Conventional Organic	27.71 ± 0.50 ^a^ 28.33 ± 1.25 ^a^	23.64 ± 0.48 ^a^ 24.12 ± 0.80 ^a^	2.88 ± 0.03 ^a^ 2.91 ± 0.02 ^a^	12.33 ± 0.60 ^a^ 14.99 ± 0.06 ^b^	45.14 ± 0.13 ^a^ 45.11 ± 0.08 ^a^	34.55 ± 0.22 ^a^ 34.57 ± 0.06 ^a^	51.34 ± 0.16 ^a^ 51.39 ± 0.13 ^a^
Saragolla	Conventional Organic	28.74 ± 1.26 ^a^ 28.24 ± 0.85 ^a^	23.84 ± 0.25 ^a^ 24.14 ± 1.12 ^a^	2.22 ± 0.03 ^a^ 2.20 ± 0.03 ^a^	7.63 ± 0.03 ^a^ 7.64 ± 0.02 ^a^	64.53 ± 0.08 ^a^ 64.47 ± 0.05 ^a^	33.84 ± 1.01 ^a^ 33.14 ± 0.07 ^a^	54.57 ± 0.09 ^a^ 54.51 ± 0.09 ^a^
Chili	Conventional Organic	27.93 ± 0.32 ^a^ 28.68 ± 0.66 ^a^	24.80 ± 0.71 ^a^ 25.86 ± 0.81 ^a^	2.45 ± 0.67 ^a^ 3.70 ± 0.02 ^b^	17.16 ± 0.07 ^a^ 17.20 ± 0.01 ^a^	57.96 ± 0.19 ^a^ 68.13 ± 0.22 ^b^	33.22 ± 0.08 ^a^ 36.60 ± 0.59 ^b^	50.46 ± 0.14 ^a^ 50.50 ± 0.08 ^a^
Biskri	Conventional Organic	28.30 ± 0.81 ^a^ 28.30 ± 0.69 ^a^	23.73 ± 0.90 ^a^ 26.04 ± 0.43 ^b^	2.26 ± 0.02 ^a^ 2.68 ± 0.38 ^b^	13.36 ± 0.04 ^a^ 13.36 ± 0.03 ^a^	46.13 ± 0.03 ^a^ 84.36 ± 0.34 ^b^	32.67 ± 2.14 ^a^ 32.35 ± 1.51 ^a^	52.17 ± 0.08 ^a^ 52.14 ± 0.07 ^a^
Mahmoudi	Conventional Organic	27.73 ± 0.72 ^a^ 27.81 ± 1.34 ^a^	24.81 ± 1,08 ^a^ 24.15 ± 0.43 ^a^	3.48 ± 0.03 ^a^ 3.47 ± 0.03 ^a^	6.22 ± 0.02 ^a^ 13.60 ± 0.17 ^b^	43.49 ± 0.07 ^a^ 74.06 ± 1.71 ^b^	33.32 ± 0.44 ^a^ 33.05 ± 0.15 ^a^	47.64 ± 0.66 ^a^ 49.23 ± 0.15 ^b^
Jenah Khotifa	Conventional Organic	27.39 ± 0.70 ^a^ 28.17 ± 0.36 ^a^	24.19 ± 0.19 ^a^ 24.12 ± 0.43 ^a^	3.35 ± 0.03 ^a^ 3.38 ± 0.02 ^a^	9.03 ± 0.99 ^a^ 8.32 ± 0.65 ^a^	48.78 ± 0.08 ^a^ 53.69 ± 1.37 ^b^	35.17 ± 1.34 ^a^ 34.64 ± 0.06 ^a^	50.64 ± 0.12 ^a^ 50.69 ± 0.09 ^a^

HMW: high molecular weight glutenin, and LMW: low molecular weight glutenin. Values are expressed as mean ± standard deviation (*n* = 3). Different letters within the same column for a given variety indicate statistically significant differences (*p* ≤ 0.05, Tukey’s test).

**Table 4:** Wet and dry gluten contents of (g/100 g dm) and gluten index (%) in whole semolina of wheat varieties grown under conventional and organic farming systems.

Wheat Variety	Farming System	Wet Gluten	Dry Gluten	Gluten Index
Karim	Conventional Organic	34.04 ± 0.03 ^b^ 30.86 ± 0.02 ^a^	14.83 ± 0.10 ^a^ 16.23 ± 0.32 ^b^	86.00 ± 2.79 ^b^ 66.00 ± 0.44 ^a^
Razzak	Conventional Organic	33.16 ± 0.25 ^b^ 31.95 ± 0.17 ^a^	12.87 ± 0.06 ^a^ 18.50 ± 0.50 ^b^	84.90 ± 0.36 ^b^ 65.03 ± 0.64 ^a^
Khiar	Conventional Organic	32.44 ± 0.23 ^b^ 29.30 ± 0.00 ^a^	10.72 ± 0.10 ^a^ 9.17 ± 0.06 ^a^	51.97 ± 1.85 ^a^ 45.03 ± 0.06 ^a^
Maali	Conventional Organic	34.72 ± 0.20 ^b^ 33.63 ± 0.00 ^a^	14.50 ± 0.40 ^b^ 11.60 ± 0.13 ^a^	55.77 ± 0.86 ^b^ 50.07 ± 0.31 ^a^
Salim	Conventional Organic	32.41 ± 0.10 ^b^ 28.21 ± 0.00 ^a^	14.27 ± 0.15 ^b^ 8.90 ± 0.10 ^a^	65.30 ± 0.90 ^b^ 56.13 ± 1.21 ^a^
INRAT 100	Conventional Organic	33.82 ± 0.09 ^b^ 32.94 ± 0.23 ^a^	17.67 ± 0.25 ^b^ 13.10 ± 0.10 ^a^	68.63 ± 2.02 ^a^ 70.17 ± 1.00 ^a^
Dhahbi	Conventional Organic	34.97 ± 0.17 ^b^ 33.44 ± 0.18 ^a^	18.58 ± 0.20 ^b^ 17.10 ± 0.36 ^a^	83.60 ± 1.31 ^b^ 66.83 ± 0.85 ^a^
Monastir	Conventional Organic	33.14 ± 0.24 ^b^ 32.04 ± 0.22 ^a^	12.97 ± 0.12 ^b^ 10.83 ± 0.21 ^a^	68.60 ± 1.39 ^b^ 57.47 ± 0.84 ^a^
Saragolla	Conventional Organic	33.46 ± 0.21 ^b^ 32.34 ± 0.21 ^a^	13.93 ± 0.49 ^b^ 11.93 ± 0.67 ^a^	70.80 ± 0.60 ^a^ 69.93 ± 1.39 ^a^
Chili	Conventional Organic	32.04 ± 0.00 ^a^ 34.02 ± 0.01 ^b^	13.77 ± 0.25 ^a^ 16.03 ± 0.32 ^b^	72.10 ± 0.79 ^a^ 83.50 ± 0.50 ^b^
Biskri	Conventional Organic	32.15 ± 0.04 ^a^ 33.86 ± 0.10 ^b^	13.73 ± 0.31 ^a^ 19.13 ± 0.72 ^b^	69.37 ± 1.15 ^a^ 71.50 ± 0.20 ^a^
Mahmoudi	Conventional Organic	32.03 ± 0.12 ^a^ 34.77 ± 0.09 ^b^	16.30 ± 0.26 ^a^ 15.87 ± 0.42 ^a^	68.70 ± 0.72 ^a^ 71.53 ± 1.11 ^b^
Jenah Khotifa	Conventional Organic	32.00 ± 0.08 ^a^ 33.83 ± 0.03 ^b^	13.60 ± 0.26 ^a^ 18.67 ± 0.15 ^b^	65.17 ± 0.59 ^a^ 76.07 ± 0.87 ^b^

Values are expressed as mean ± standard deviation (*n* = 3). Different letters within the same column for a given variety indicate statistically significant differences (*p* ≤ 0.05, Tukey’s test).

**Table 5:** Content of essential amino acids (g/100 g dm) in whole semolina of wheat varieties grown under conventional and organic farming systems.

Wheat Variety	Farming System	Val	Leu	Ile	Lys	His	Phe	Thr	Met	Total
Karim	Conventional	0.68 ± 0.01 ^b^	0.95 ± 0.00 ^b^	0.66 ± 0.02 ^b^	0.70 ± 0.02 ^b^	0.48 ± 0.01 ^b^	0.74 ± 0.02 ^b^	0.58 ± 0.01 ^b^	0.26 ± 0.02 ^b^	5.05 ± 0.04 ^b^
	Organic	0.53 ± 0.02 ^a^	0.85 ± 0.02 ^a^	0.40 ± 0.01 ^a^	0.35 ± 0.00 ^a^	0.36 ± 0.01 ^a^	0.58 ± 0.01 ^a^	0.36 ± 0.02 ^a^	0.14 ± 0.01 ^a^	3.57 ± 0.04 ^a^
Razzak	Conventional	0.80 ± 0.02 ^b^	0.96 ± 0.02 ^b^	0.57 ± 0.02 ^a^	0.58 ± 0.00 ^b^	0.37 ± 0.02 ^a^	0.68 ± 0.02 ^b^	0.50 ± 0.01 ^b^	0.24 ± 0.02 ^b^	4.70 ± 0.04 ^b^
	Organic	0.52 ± 0.01 ^a^	0.84 ± 0.02 ^a^	0.39 ± 0.01 ^b^	0.34 ± 0.00 ^a^	0.38 ± 0.02 ^a^	0.57 ± 0.00 ^a^	0.36 ± 0.01 ^a^	0.13 ± 0.01 ^a^	3.53 ± 0.03 ^a^
Khiar	Conventional	0.52 ± 0.02 ^b^	0.82 ± 0.01 ^b^	0.37 ± 0.02 ^a^	0.32 ± 0.01 ^a^	0.29 ± 0.00 ^a^	0.53 ± 0.00 ^b^	0.34 ± 0.01 ^a^	0.13 ± 0.01 ^a^	3.32 ± 0.03 ^b^
	Organic	0.47 ± 0.01 ^a^	0.76 ± 0.01 ^a^	0.34 ± 0.02 ^a^	0.30 ± 0.01 ^a^	0.28 ± 0.00 ^a^	0.48 ± 0.01 ^a^	0.32 ± 0.02 ^a^	0.11 ± 0.01 ^a^	3.06 ± 0.06 ^a^
Maali	Conventional	0.48 ± 0.02 ^a^	0.78 ± 0.01 ^b^	0.36 ± 0.02 ^a^	0.31 ± 0.01 ^a^	0.30 ± 0.02 ^a^	0.52 ± 0.01 ^b^	0.38 ± 0.01 ^b^	0.16 ± 0.00 ^b^	3.29 ± 0.04 ^b^
	Organic	0.47 ± 0.02 ^a^	0.72 ± 0.01 ^a^	0.34 ± 0.01 ^a^	0.30 ± 0.00 ^a^	0.29 ± 0.01 ^a^	0.46 ± 0.02 ^a^	0.31 ± 0.00 ^a^	0.11 ± 0.00 ^a^	3.00 ± 0.02 ^a^
Salim	Conventional	0.54 ± 0.02 ^a^	0.85 ± 0.00 ^b^	0.42 ± 0.00 ^b^	0.34 ± 0.02 ^a^	0.33 ± 0.00 ^a^	0.59 ± 0.01 ^b^	0.37 ± 0.01 ^b^	0.15 ± 0.02 ^a^	3.59 ± 0.04 ^b^
	Organic	0.49 ± 0.01 ^b^	0.77 ± 0.02 ^a^	0.34 ± 0.01 ^a^	0.32 ± 0.01 ^a^	0.35 ± 0.02 ^b^	0.51 ± 0.02 ^a^	0.33 ± 0.02 ^a^	0.11 ± 0.02 ^a^	3.22 ± 0.02 ^a^
INRAT 100	Conventional	0.58 ± 0.02 ^a^	0.92 ± 0.02 ^a^	0.45 ± 0.02 ^a^	0.36 ± 0.01 ^a^	0.35 ± 0.02 ^a^	0.61 ± 0.01 ^a^	0.40 ± 0.01 ^a^	0.16 ± 0.01 ^a^	3.83 ± 0.05 ^a^
	Organic	0.61 ± 0.00 ^b^	0.93 ± 0.00 ^a^	0.46 ± 0.01 ^a^	0.38 ± 0.02 ^a^	0.46 ± 0.00 ^b^	0.67 ± 0.01 ^b^	0.41 ± 0.02 ^a^	0.17 ± 0.01 ^a^	4.09 ± 0.01 ^b^
Dhahbi	Conventional	0.59 ± 0.02 ^b^	0.98 ± 0.01 ^a^	0.50 ± 0.01 ^b^	0.37 ± 0.01 ^b^	0.36 ± 0.01 ^b^	0.66 ± 0.00 ^a^	0.41 ± 0.01 ^b^	0.17 ± 0.02 ^a^	4.04 ± 0.05 ^b^
	Organic	0.55 ± 0.00 ^a^	0.87 ± 0.02 ^a^	0.42 ± 0.02 ^a^	0.34 ± 0.01 ^a^	0.31 ± 0.00 ^a^	0.65 ± 0.01 ^a^	0.39 ± 0.00 ^a^	0.19 ± 0.01 ^a^	3.72 ± 0.01 ^a^
Monastir	Conventional	0.57 ± 0.01 ^b^	0.91 ± 0.01 ^b^	0.44 ± 0.02 ^b^	0.35 ± 0.00 ^b^	0.33 ± 0.02 ^a^	0.60 ± 0.02 ^b^	0.38 ± 0.01 ^a^	0.16 ± 0.01 ^b^	3.74 ± 0.04 ^b^
	Organic	0.51 ± 0.00 ^a^	0.79 ± 0.01 ^a^	0.36 ± 0.01 ^a^	0.33 ± 0.00 ^a^	0.30 ± 0.02 ^a^	0.52 ± 0.00 ^a^	0.34 ± 0.01 ^b^	0.12 ± 0.01 ^a^	3.27 ± 0.01 ^a^
Saragolla	Conventional	0.60 ± 0.01 ^b^	0.90 ± 0.01 ^a^	0.42 ± 0.00 ^a^	0.36 ± 0.00 ^a^	0.35 ± 0.02 ^a^	0.62 ± 0.02 ^a^	0.40 ± 0.02 ^a^	0.19 ± 0.02 ^a^	3.84 ± 0.01 ^a^
	Organic	0.57 ± 0.01 ^a^	0.89 ± 0.00 ^a^	0.44 ± 0.02 ^b^	0.36 ± 0.00 ^a^	0.34 ± 0.01 ^a^	0.62 ± 0.00 ^a^	0.40 ± 0.02 ^a^	0.17 ± 0.00 ^a^	3.79 ± 0.06 ^a^
Chili	Conventional	0.49 ± 0.02 ^a^	0.75 ± 0.01 ^a^	0.33 ± 0.02 ^a^	0.29 ± 0.00 ^a^	0.30 ± 0.01 ^a^	0.46 ± 0.00 ^a^	0.30 ± 0.02 ^a^	0.11 ± 0.02 ^a^	3.03 ± 0.01 ^a^
	Organic	0.56 ± 0.02 ^b^	0.87 ± 0.00 ^b^	0.43 ± 0.01 ^b^	0.35 ± 0.02 ^b^	0.33 ± 0.01 ^b^	0.70 ± 0.01 ^b^	0.38 ± 0.02 ^b^	0.15 ± 0.01 ^b^	3.77 ± 0.07 ^b^
Biskri	Conventional	0.55 ± 0.02 ^a^	0.78 ± 0.02 ^a^	0.41 ± 0.01 ^a^	0.33 ± 0.01 ^a^	0.30 ± 0.01 ^a^	0.55 ± 0.01 ^a^	0.34 ± 0.01 ^a^	0.18 ± 0.02 ^b^	3.44 ± 0.00 ^a^
	Organic	0.56 ± 0.01 ^a^	0.89 ± 0.01 ^b^	0.43 ± 0.01 ^a^	0.35 ± 0.00 ^b^	0.54 ± 0.00 ^b^	0.76 ± 0.02 ^b^	0.38 ± 0.01 ^b^	0.15 ± 0.00 ^a^	4.06 ± 0.05 ^b^
Mahmoudi	Conventional	0.48 ± 0.01 ^a^	0.77 ± 0.02 ^a^	0.35 ± 0.00 ^a^	0.30 ± 0.01 ^a^	0.28 ± 0.02 ^a^	0.49 ± 0.01 ^a^	0.32 ± 0.01 ^a^	0.12 ± 0.01 ^a^	3.11 ± 0.01 ^a^
	Organic	0.60 ± 0.01 ^b^	0.94 ± 0.00 ^b^	0.46 ± 0.00 ^b^	0.37 ± 0.02 ^b^	0.47 ± 0.01 ^b^	0.62 ± 0.02 ^b^	0.41 ± 0.02 ^b^	0.16 ± 0.02 ^b^	4.03 ± 0.23 ^b^
Jenah	Conventional	0.49 ± 0.02 ^a^	0.76 ± 0.01 ^a^	0.34 ± 0.02 ^a^	0.32 ± 0.02 ^a^	0.31 ± 0.01 ^a^	0.55 ± 0.02 ^a^	0.33 ± 0.01 ^a^	0.12 ±0.01 ^a^	3.22 ± 0.05 ^a^
Khotifa	Organic	0.53 ± 0.02 ^b^	0.85 ± 0.02 ^b^	0.40 ± 0.00 ^b^	0.33 ± 0.02 ^a^	0.32 ± 0.00 ^a^	0.64 ± 0.00 ^b^	0.38 ± 0.01 ^b^	0.18 ± 0.00 ^b^	3.63 ± 0.07 ^b^

Values are expressed as mean ± standard deviation (*n* = 3). Different letters within the same column for a given variety indicate statistically significant differences (*p* ≤ 0.05, Tukey’s test). Val: valine; Leu: leucine; Ile: isoleucine; Lys: lysine; His: histidine; Phe: phenilalanine; Thr: threonine; and Met: metionine.

**Table 6:** Content of non-essential amino acids (g/100 dm) in whole semolina of wheat varieties grown under conventional and organic farming systems.

Wheat Variety	Farming System	Ala	Gly	Pro	Asp	Glu	Arg	Tyr	Ser	Cys	Total
Karim	Conventional	0.61 ± 0.01 ^b^	0.58 ± 0.01 ^b^	1.53 ± 0.02 ^b^	0.76 ± 0.00 ^b^	6.15 ± 0.02 ^b^	0.74 ± 0.01 ^b^	0.42 ± 0.01 ^b^	0.80 ± 0.00 ^b^	0.30 ± 0.00 ^a^	11.89 ± 0.02 ^b^
	Organic	0.44 ± 0.01 ^a^	0.50 ± 0.01 ^a^	1.32 ± 0.01 ^a^	0.66 ± 0.01 ^a^	4.60 ± 0.02 ^a^	0.58 ± 0.02 ^a^	0.32 ± 0.02 ^a^	0.66 ± 0.02 ^a^	0.33 ± 0.00 ^b^	9.41 ± 0.04 ^a^
Razzak	Conventional	0.58 ± 0.01 ^b^	0.75 ± 0.00 ^b^	1.49 ± 0.01 ^b^	0.85 ± 0.00 ^b^	6.22 ± 0.02 ^b^	0.83 ± 0.02 ^b^	0.41 ± 0.01 ^b^	0.82 ± 0.01 ^b^	0.25 ± 0.02 ^a^	12.20 ± 0.04 ^b^
	Organic	0.43 ± 0.01 ^a^	0.48 ± 0.01 ^a^	1.27 ± 0.01 ^a^	0.64 ± 0.02 ^a^	4.65 ± 0.02 ^a^	0.59 ± 0.02 ^a^	0.33 ± 0.01 ^a^	0.67 ± 0.01 ^a^	0.26 ± 0.02 ^a^	9.32 ± 0.00 ^a^
Khiar	Conventional	0.41 ± 0.00 ^b^	0.46 ± 0.01 ^a^	1.00 ± 0.02 ^a^	0.59 ± 0.01 ^b^	4.10 ± 0.00 ^b^	0.51 ± 0.00 ^a^	0.27 ± 0.01 ^a^	0.59 ± 0.01 ^b^	0.17 ± 0.02 ^a^	8.10 ± 0.02 ^b^
	Organic	0.39 ± 0.01 ^a^	0.44 ± 0.02 ^a^	0.98 ± 0.01 ^a^	0.53 ± 0.02 ^a^	3.95 ± 0.01 ^a^	0.51 ± 0.02 ^a^	0.27 ± 0.02 ^a^	0.57 ± 0.00 ^a^	0.20 ± 0.01 ^b^	7.84 ± 0.05 ^a^
Maali	Conventional	0.39 ± 0.01 ^a^	0.44 ± 0.01 ^a^	1.02 ± 0.01 ^a^	0.57 ± 0.02 ^a^	4.15 ± 0.01 ^b^	0.52 ± 0.02 ^a^	0.28 ± 0.00 ^b^	0.58 ± 0.00 ^a^	0.21 ± 0.01 ^a^	8.16 ± 0.07 ^b^
	Organic	0.39 ± 0.00 ^a^	0.45 ± 0.00 ^a^	1.03 ± 0.01 ^a^	0.58 ± 0.01 ^a^	4.00 ± 0.02 ^a^	0.49 ± 0.01 ^a^	0.26 ± 0.01 ^a^	0.57 ± 0.00 ^a^	0.23 ± 0.02 ^a^	8.00 ± 0.03 ^a^
Salim	Conventional	0.44 ± 0.02 ^a^	0.50 ± 0.02 ^b^	1.18 ± 0.02 ^b^	0.63 ± 0.01 ^a^	4.55 ± 0.01 ^b^	0.61 ± 0.01 ^b^	0.34 ± 0.00 ^b^	0.69 ± 0.02 ^b^	0.24 ± 0.01 ^a^	9.18 ± 0.01 ^b^
	Organic	0.42 ± 0.02 ^a^	0.47 ± 0.01 ^a^	1.12 ± 0.02 ^a^	0.60 ± 0.02 ^a^	4.25 ± 0.02 ^a^	0.57 ± 0.02 ^a^	0.31 ± 0.01 ^a^	0.64 ± 0.00 ^a^	0.26 ± 0.02 ^a^	8.64 ± 0.02 ^a^
INRAT 100	Conventional	0.49 ± 0.01 ^a^	0.55 ± 0.01 ^a^	1.47 ± 0.01 ^a^	0.72 ± 0.02 ^a^	5.90 ± 0.01 ^a^	0.70 ± 0.02 ^a^	0.39 ± 0.01 ^a^	0.76 ± 0.01 ^a^	0.28 ± 0.02 ^a^	11.26 ± 0.07 ^a^
	Organic	0.52 ± 0.02 ^b^	0.59 ± 0.00 ^b^	1.48 ± 0.02 ^a^	0.76 ± 0.00 ^b^	6.00 ± 0.00 ^b^	0.71 ± 0.01 ^a^	0.40 ± 0.02 ^a^	0.78 ± 0.01 ^a^	0.29 ± 0.02 ^a^	11.53 ± 0.06 ^b^
Dhahbi	Conventional	0.50 ± 0.02 ^b^	0.57 ± 0.01 ^b^	1.52 ± 0.01 ^b^	0.74 ± 0.02 ^b^	6.05 ± 0.01 ^b^	0.72 ± 0.01 ^b^	0.41 ± 0.02 ^b^	0.79 ± 0.02 ^b^	0.29 ± 0.00 ^a^	11.59 ± 0.01 ^b^
	Organic	0.46 ± 0.01 ^a^	0.52 ± 0.01 ^a^	1.22 ± 0.01 ^a^	0.69 ± 0.01 ^a^	4.80 ± 0.01 ^a^	0.61 ± 0.02 ^a^	0.35 ± 0.02 ^a^	0.68 ± 0.01 ^a^	0.29 ± 0.02 ^a^	9.62 ± 0.03 ^a^
Monastir	Conventional	0.47 ± 0.02 ^b^	0.54 ± 0.01 ^b^	1.36 ± 0.00 ^b^	0.70 ± 0.01 ^b^	5.40 ± 0.01 ^b^	0.63 ± 0.01 ^b^	0.36 ± 0.01 ^b^	0.72 ± 0.02 ^b^	0.22 ± 0.02 ^a^	10.40 ± 0.07 ^b^
	Organic	0.43 ± 0.01 ^a^	0.49 ± 0.02 ^a^	1.08 ± 0.01 ^a^	0.62 ± 0.01 ^a^	4.20 ± 0.01 ^a^	0.56 ± 0.00 ^a^	0.30 ± 0.01 ^a^	0.63 ± 0.01 ^a^	0.25 ± 0.01 ^b^	8.56 ± 0.01 ^a^
Saragolla	Conventional	0.47 ± 0.00 ^a^	0.52 ± 0.00 ^a^	1.37 ± 0.00 ^b^	0.69 ± 0.02 ^a^	5.55 ± 0.02 ^b^	0.66 ± 0.02 ^a^	0.38 ± 0.02 ^a^	0.75 ± 0.02 ^a^	0.26 ± 0.02 ^a^	10.65 ± 0.06 ^b^
	Organic	0.48 ± 0.02 ^a^	0.53 ± 0.01 ^a^	1.34 ± 0.01 ^a^	0.71 ± 0.01 ^a^	5.50 ± 0.01 ^a^	0.65 ± 0.01 ^a^	0.37 ± 0.00 ^a^	0.74 ± 0.02 ^a^	0.27 ± 0.01 ^a^	10.59 ± 0.00 ^a^
Chili	Conventional	0.38 ± 0.01 ^a^	0.44 ± 0.01 ^a^	1.05 ± 0.02 ^a^	0.56 ± 0.00 ^a^	4.05 ± 0.01 ^a^	0.48 ± 0.02 ^a^	0.27 ± 0.01 ^a^	0.56 ± 0.00 ^a^	0.21 ± 0.02 ^a^	8.00 ± 0.08 ^a^
	Organic	0.45 ± 0.00 ^b^	0.51 ± 0.01 ^b^	1.15 ± 0.01 ^b^	0.65 ± 0.01 ^b^	4.50 ± 0.00 ^b^	0.60 ± 0.02 ^b^	0.33 ± 0.01 ^b^	0.68 ± 0.02 ^b^	0.22 ± 0.01 ^a^	9.09 ± 0.03 ^b^
Biskri	Conventional	0.42 ± 0.01 ^a^	0.48 ± 0.00 ^a^	1.10 ± 0.00 ^a^	0.62 ± 0.01 ^a^	4.35 ± 0.02 ^a^	0.54 ± 0.01 ^a^	0.29 ± 0.02 ^a^	0.61 ± 0.01 ^a^	0.18 ± 0.01 ^a^	8.59 ± 0.06 ^a^
	Organic	0.46 ± 0.00 ^b^	0.53 ± 0.01 ^b^	1.39 ± 0.00 ^b^	0.68 ± 0.02 ^b^	5.45 ± 0.01 ^b^	0.64 ± 0.02 ^b^	0.37 ± 0.01 ^b^	0.73 ± 0.00 ^b^	0.26 ± 0.01 ^b^	10.51 ± 0.05 ^b^
Mahmoudi	Conventional	0.40 ± 0.01 ^a^	0.45 ± 0.01 ^a^	0.95 ± 0.02 ^a^	0.55 ± 0.02 ^a^	5.37 ± 0.02 ^a^	0.50 ± 0.00 ^a^	0.26 ± 0.02 ^a^	0.56 ± 0.02 ^a^	0.16 ± 0.02 ^a^	9.20 ± 0.03 ^a^
	Organic	0.50 ± 0.01 ^b^	0.57 ± 0.00 ^b^	1.55 ± 0.00 ^b^	0.74 ± 0.02 ^b^	5.85 ± 0.02 ^b^	0.69 ± 0.02 ^b^	0.38 ± 0.02 ^b^	0.75 ± 0.00 ^b^	0.23 ± 0.02 ^b^	11.26 ± 0.00 ^b^
Jenah	Conventional	0.41 ± 0.00 ^a^	0.46 ± 0.02 ^a^	1.14 ± 0.02 ^a^	0.60 ± 0.01 ^a^	4.40 ± 0.01 ^a^	0.55 ± 0.02 ^a^	0.30 ± 0.01 ^a^	0.62 ± 0.01 ^a^	0.22 ± 0.01 ^a^	8.70 ± 0.00 ^a^
Khotifa	Organic	0.45 ± 0.01 ^a^	0.51 ± 0.02 ^b^	1.26 ± 0.01 ^b^	0.67 ± 0.00 ^b^	4.85 ± 0.01 ^b^	0.60 ± 0.01 ^b^	0.34 ± 0.01 ^b^	0.67 ± 0.02 ^b^	0.23 ± 0.01 ^a^	9.58 ± 0.02 ^b^

Values are expressed as mean ± standard deviation (*n* = 3). Different letters within the same column for a given variety indicate statistically significant differences (*p* ≤ 0.05, Tukey’s test). Ala: alanine; Gly: glicine; Pro: proline; Asp: aspartic acid; Glu: glutamic acid; Arg: arginine; Tyr: tyrosine; Ser: serine; and Cys: cysteine.

**Table 7:** Content of total starch, amylose and amylopectin (g/100 g dm) in whole semolina of wheat varieties grown under conventional and organic farming systems.

Wheat Variety	Farming System	Total Starch	Amylose	Amylopectin
Karim	Conventional Organic	87.39 ± 2.32 ^b^ 52.51 ± 2.43 ^a^	23.91 ± 1.45 ^a^ 27.80 ± 1.68 ^b^	76.09 ± 4.63 ^a^ 72.20 ± 4.61 ^a^
Razzak	Conventional Organic	62.00 ± 2.18 ^b^ 56.67 ± 2.17 ^a^	22.76 ± 1.50 ^a^ 30.56 ± 1.14 ^b^	77.24 ± 2.68 ^b^ 69.44 ± 4.62 ^a^
Khiar	Conventional Organic	52.95 ± 1.77 ^a^ 51.27 ± 1.94 ^a^	30.31 ± 0.83 ^a^ 33.61 ± 0.78 ^b^	69.69 ± 3.53 ^a^ 66.39 ± 3.99 ^a^
Maali	Conventional Organic	63.23 ± 2.86 ^b^ 51.97 ± 1.34 ^a^	26.09 ± 1.82 ^a^ 38.50 ± 0.89 ^b^	73.91 ± 3.65 ^b^ 61.50 ± 3.07 ^a^
Salim	Conventional Organic	76.56 ± 2.64 ^b^ 54.85 ± 1.79 ^a^	29.28 ± 0.75 ^b^ 26.45 ± 1.80 ^a^	70.72 ± 3.24 ^a^ 73.55 ± 3.26 ^a^
INRAT 100	Conventional Organic	70.64± 1.39 ^b^ 54.20 ± 3.31 ^a^	22.72 ± 1.15 ^a^ 30.83 ± 1.36 ^b^	77.28 ± 2.82 ^b^ 69.17 ± 1.84 ^a^
Dhahbi	Conventional Organic	84.67 ± 3.00 ^b^ 68.15 ± 3.33 ^a^	23.93 ± 2.94 ^a^ 40.81 ± 1.20 ^b^	76.07 ± 2.50 ^b^ 59.19 ± 4.31 ^a^
Monastir	Conventional Organic	87.53 ± 3.85 ^b^ 62.20 ± 2.61 ^a^	35.62 ± 2.54 ^a^ 30.69 ± 1.21 ^a^	64.38 ± 3.87 ^a^ 69.31 ± 2.29 ^b^
Saragolla	Conventional Organic	57.86 ± 1.45 ^b^ 50.67 ± 1.70 ^a^	23.23 ± 0.77 ^a^ 46.99 ± 1.24 ^b^	76.77 ± 2.40 ^b^ 53.01 ± 2.15 ^a^
Chili	Conventional Organic	50.46 ± 0.79 ^b^ 47.25 ± 1.31 ^a^	22.34 ± 0.98 ^a^ 26.11 ± 1.22 ^b^	77.66 ± 1.29 ^b^ 73.89 ± 2.94 ^a^
Biskri	Conventional Organic	52.59 ± 1.13 ^b^ 49.23 ± 1.90 ^a^	21.76 ± 2.03 ^a^ 24.06 ± 0.54 ^b^	78.24 ± 2.57 ^a^ 75.94 ± 2.19 ^a^
Mahmoudi	Conventional Organic	51.75 ± 1.54 ^b^ 46.92 ± 1.55 ^a^	16.03 ± 1.37 ^a^ 19.76 ± 0.55 ^b^	83.97 ± 1.79 ^b^ 80.24 ± 1.87 ^a^
Jenah Khotifa	Conventional Organic	50.29 ± 2.02 ^a^ 49.40 ± 0.78 ^a^	20.90 ± 1.38 ^a^ 23.46 ± 1.06 ^b^	79.10 ± 2.62 ^a^ 76.54 ± 3.31 ^a^

Values are expressed as mean ± standard deviation (*n* = 3). Different letters within the same column indicate statistically significant differences (*p* ≤ 0.05, Tukey’s test).

**Table 8:** Total and immunogenic peptide profiles identified in two durum wheat varieties (Chili and INRAT100) grown under conventional (C) and organic (O) farming systems.

	Chili C	Chili O	INRAT100 C	INRAT100 O
Total peptides	1665	998	851	622
Immunogenic peptides	139	47	57	33
Total PSMs	16,317	4101	3939	3145
Immunogenic PSMs	2841	192	298	175
Immunogenic peptides (%)	8.35	4.71	6.70	5.31
Immunogenic abundance (%)	17.41	4.68	7.57	5.56

PSM: Peptide spectrum matches.

## Data Availability

The original contributions presented in this study are included in the article/Supplementary Materials. Further inquiries can be directed to the corresponding author.

## Supplementary Materials

The following supporting information can be downloaded at: https://www.mdpi.com/article/10.3390/foods15071121/s1, Table S1. Peptides identified by LC–MS/MS and immunogenic epitope mapping after INFOGEST 2.0 digestion of Chilli durum wheat under conventional cultivation. Table S2. Peptides identified by LC–MS/MS and immunogenic epitope mapping after INFOGEST 2.0 digestion of Chilli durum wheat under organic cultivation. Table S3. Peptides identified by LC–MS/MS and immunogenic epitope mapping after INFOGEST 2.0 digestion of INRAT100 durum wheat under conventional cultivation. Table S4. Peptides identified by LC–MS/MS and immunogenic epitope mapping after INFOGEST 2.0 digestion of INRAT100 durum wheat under organic cultivation. Figure S1. Relative proportion of immunogenic peptides and their abundance in Chili and INRAT100 durum wheat samples under conventional and organic cultivation.

## Abbreviations

The following abbreviations are used in this manuscript:

AA	Amino acids
HBr	Hydrogen bromide
HMW	High molecular weight glutenin
LMW	Low molecular weight glutenin
PVDF	Polyvinylidene Fluoride
PSMs	Peptide-spectrum matches

## Appendix A

**Table A1:** Mean square values from the combined analysis of variance (ANOVA) for protein content, wet gluten, gluten index, dry gluten, albumin, globulin, gliadin, glutenin, ω-, αβ-, and γ-gliadin fractions in durum wheat varieties grown under organic and conventional farming systems.

Source of Variation	df	Protein Content	Wet Gluten	Gluten Index	Dry Gluten	Albumin	Globulin	Gliadin	Glutenin	ω	αβ	γ
Corrected Model	27	2.18	6.83	309.61	23.41	0.39	1.02	0.02	0.01	1.14	37.04	598.67
Constant	1	14,300.68	83,676.91	357,808.11	16,208.08	61,391.91	45,938.26	15.01	11.12	570.57	7665.78	256,613.73
Replication	2	0.00	0.16	4.08	0.16	0.56	0.12	0.00	0.00	0.06	0.32	4.52
System	1	1.44 ***	9.74 ***	449.76 ***	0.05 ^ns^	0.51 ^ns^	1.58 ^ns^	0.06 ***	0.04 ***	0.71 ***	13.39 ***	355.88 ***
Variety	12	3.01 ***	7.40 ***	488.27 ***	32.00 ***	0.38 ^ns^	1.20 *	0.05 ***	0.02 ***	2.27 ***	70.91 ***	498.65 ***
System * Variety	12	1.78 ***	7.13 ***	170.20 ***	20.63 ***	0.35 ^ns^	0.94 ^ns^	0.00 ***	0.00 *	0.22 ***	11.25 ***	817.95 ***
Error	50	0.00	0.02	1.14	0.10	0.58	0.56	0.00	0.00	0.03	0.18	3.25
Total	78											
Corrected Total	77											
**CV**		**6.45**	**4.63**	**15.12**	**20.10**	**2.55**	**3.46**	**0.00**	**0.00**	**15.70**	**36.49**	**25.27**
**R^2^**		**1.00**	**1.00**	**0.99**	**0.99**	**0.27**	**0.50**	**0.99**	**0.93**	**0.95**	**0.99**	**0.99**

ns, *, and *** indicate a non-significant effect and significant effects at the 5% and 0.1% probability levels, respectively.

**Table A2:** Mean square values of non-essential and essential amino acid contents in durum wheat varieties grown under organic and conventional farming systems.

**Non-essential amino acid**											
	**df**	**Alanine**	**Glycine**	**Proline**	**Aspartic acid**	**Glutamic acid**	**Arginine**	**Tyrosine**	**Serine**	**Cysteine**	**Total**
Corrected Model	27	0.01	0.01	0.11	0.02	2.07	0.02	0.01	0.02	0.01	5.07
Constant	1	16.04	20.50	120.90	33.78	1915.36	28.77	8.75	35.82	4.59	7319.83
Replication	2	0.00	0.00	0.00	0.00	0.00	0.00	0.00	0.00	0.00	0.00
System	1	0.00 ***	0.00 ***	0.00 ^ns^	0.00 ^ns^	1.53 ***	0.00 ***	0.00 ^ns^	0.00 *	0.01 ***	1.82 ***
Variety	12	0.01 ***	0.01 ***	0.14 ***	0.02 ***	2.46 ***	0.03 ***	0.01 ***	0.02 ***	0.01 ***	7.40 ***
System * Variety	12	0.01 ***	0.01 ***	0.09 ***	0.02 ***	1.11 ***	0.02 ***	0.01 ***	0.02 ***	0.00 ***	3.86 ***
Error	50	0.00	0.00	0.00	0.00	0.00	0.00	0.00	0.00	0.00	0.00
Total	78										
Corrected Total	77										
**CV**		**12.09**	**11.65**	**15.27**	**11.36**	**15.12**	**14.05**	**14.93**	**11.85**	**18.37**	**13.63**
**R^2^**		**0.97**	**0.98**	**1.00**	**0.98**	**1.00**	**0.98**	**0.95**	**0.98**	**0.91**	**1.00**
**Essential amino acid**											
	**df**	**Valine**	**Leucine**	**Isoleucine**	**Lysine**	**Histidine**	**Phenylalanine**		**Threonine**	**Methionine**	**Total**
Corrected Model	27	0.01	0.02	0.02	0.02	0.01	0.02		0.01	0.00	0.68
Constant	1	23.73	56.36	13.53	10.09	9.51	27.29		11.13	1.88	1040.03
Replication	2	0.00	0.00	0.00	0.00	0.00	0.00		0.00	0.00	0.01
System	1	0.02 ***	0.00 ***	0.02 ***	0.03 ***	0.02 ***	0.00 ***		0.01 ***	0.01 ***	0.25 ***
Variety	12	0.02 ***	0.02 ***	0.02 ***	0.02 ***	0.01 ***	0.02 ***		0.01 ***	0.00 ***	0.75 ***
System * Variety	12	0.02 ***	0.02 ***	0.02 ***	0.02 ***	0.01 ***	0.02 ***		0.01 ***	0.00 ***	0.75 ***
Error	50	0.00	0.00	0.00	0.00	0.00	0.00		0.00	0.00	0.00
Total	78										
Corrected Total	77										
**CV**		**11.7**	**8.7**	**15.7**	**24.2**	**18.6**	**14.4**		**15.0**	**25.0**	**13.00**
**R^2^**		**0.97**	**0.98**	**0.98**	**0.99**	**0.97**	**0.98**		**0.96**	**0.92**	**1.00**

ns, * and *** indicate non-significant effects and significant effects at the 5% and 0.1% levels respectively.

**Table A3:** Mean square values from the combined analysis of variance (ANOVA) for high-molecular-weight glutenins (HMW), low-molecular-weight glutenins (LMW), total starch, amylose, and amylopectin in durum wheat varieties grown under organic and conventional farming systems.

Source of Variation	df	Total Starch	Amylose	Amylopectin
Corrected Model	27	428.98	137.90	141.15
Constant	1	274,792.88	59,570.84	408,462.07
Replication	2	3.26	2.43	46.22
System	1	2687.99 ***	752.31 ***	752.37 ***
Variety	12	573.66 ***	156.37 ***	156.34 ***
System * Variety	12	167.02 ***	90.80 ***	90.85 ***
Error	50	4.60	1.95	8.13
Total	78			
Corrected Total	77			
**CV**		**7.75**	**7.06**	**11.23**
**R^2^**		**0.98**	**0.97**	**0.90**

*** indicate a significant effect at the 0.1% level.

## References

[1] Mäder P., Fliessbach A., Dubois D., Gunst L., Fried P., Niggli U. (2002). Soil fertility and biodiversity in organic farming. Science.

[2] Harasim E., Kwiatkowski C.A., Bielińska J. (2025). The influence of the farming system and forecrop on the yield and quality of spring wheat grain. Agronomy.

[3] Reganold J.P., Wachter J.M. (2016). Organic agriculture in the twenty-first century. Nat. Plants.

[4] Rached Z., Chebil A., Thabet C. (2023). Effect of farm size on sustainability dimensions: Case of durum wheat in Northern Tunisia. Sustainability.

[5] Paull J. (2024). Organic agriculture in Tunisia, Africa. Eur. J. Agric. Food Sci..

[6] Boukid F., Vittadini E., Prandi B., Mattarozzi M., Marchini M., Sforza S., Sayar R., Seo Y.W., Yacoubi I., Mejri M. (2018). Insights into a century of breeding of durum wheat in Tunisia: The properties of flours and starches isolated from landraces, old and modern genotypes. LWT.

[7] Stagnari F., Onofri A., Codianni P., Pisante M. (2013). Durum wheat varieties in N-deficient environments and organic farming: A comparison of yield, quality and stability performances. Plant Breed..

[8] Rempelos L., Kabourakis E., Leifert C. (2023). Innovative organic and regenerative agricultural production. Agronomy.

[9] Urbanavičiūtė I., Bonfiglioli L., Pagnotta M.A. (2024). Selection of durum wheat and SSR markers for organic farming in central Italy using AMMI analysis. Agronomy.

[10] Bradauskiene V., Vaiciulyte-Funk L., Martinaitiene D., Andruskiene J., Verma A.K., Lima J.P.M., Serin Y., Catassi C. (2023). Wheat consumption and prevalence of celiac disease: Correlation from a multilevel analysis. Crit. Rev. Food Sci. Nutr..

[11] Grover J., Chhuneja P., Midha V., Ghia J.E., Deka K., Mukhopadhyay C.S., Sood N., Mahajan R., Singh A., Verma R. (2019). Variable Immunogenic potential of wheat: Prospective for selection of innocuous varieties for celiac disease patients via in vitro approach. Front. Immunol..

[12] Van den Broeck H.C., de Jong H.C., Salentijn E.M.J., Dekking L., Bosch D., Hamer R.J., Gilissen L.J.W.J., van de Meer I.M., Smulders M.J.M. (2010). Presence of celiac disease epitopes in modern and old hexaploid wheat varieties: Wheat breeding may have contributed to increased prevalence of celiac disease. Theor. Appl. Genet..

[13] Cebolla A., Moreno M.L., Coto L., Sousa C. (2018). Gluten immunogenic peptides as standard for the evaluation of potential harmful prolamin content in food and human specimen. Nutrients.

[14] Mamone G., Di Stasio L., Vitale S., Picascia S., Gianfrani C. (2023). Analytical and functional approaches to assess the immunogenicity of gluten proteins. Front. Nutr..

[15] Prandi B., Tedeschi T., Folloni S., Galaverna G., Sforza S. (2017). Peptides from gluten digestion: A comparison between old and modern wheat varieties. Food Res. Int..

[16] Bartos A., Malik A., Feledyn Szewczyk B., Jończyk K., Kazimierczak R., Hallmann E., Leszczyńska J. (2025). Polyphenolic and immunometric profiling of wheat varieties: Impact of organic and conventional farming on allergenic and bioactive compounds. Molecules.

[17] Fares C., Menga V., Codianni P., Russo M., Perrone D., Suriano S., Savino M., Rascio A. (2019). Phenolic acids variability and grain quality oforganically and conventionally fertilised old wheats under a warm climate. J. Sci. Food Agric..

[18] Melios S., Ninou E., Irakli M., Tsivelika N., Sistanis J., Papathanasiou F., Didos S., Zinoviadou K., Karantonis H.C., Argiriou A. (2024). Effect of genotype, environment, and their interaction on the antioxidant properties of durum wheat: Impact of nitrogen fertilization and sowing time. Agriculture.

[19] AFNOR NF V03-050. https://www.boutique.afnor.org/Store/Preview/DisplayExtract?ProductID=55141&VersionID=6.

[20] Lookhart G., Bean S. (1995). Separation and characterization of wheat protein fractions by high-performance capillary electrophoresis. Cereal Chem..

[21] Wieser H., Antes S., Seilmeier W. (1998). Quantitative determination of gluten protein types in wheat flour by reversed-phase high-performance liquid chromatography. Cereal Chem..

[22] Bradford M.M. (1976). A rapid and sensitive method for the quantification of microgram quantities of protein utilizing the principle of protein-dye binding. Anal. Biochem..

[23] Vigni M.L., Baschieri C., Marchetti A., Cocchi M. (2013). RP-HPLC and chemometrics for wheat flour protein characterisation in an industrial bread-making process monitoring context. Food Chem..

[24] Wheat and Wheat Flour—Gluten Content—Part 2: Determination of Wet Gluten and Gluten Index by Mechanical Means.

[25] Wheat and Wheat Flour—Gluten Content—Part 3: Determination of Dry Gluten from Wet Gluten by an Oven Drying Method.

[26] Chen X., He X., Fu X., Huang Q. (2015). In vitro digestion and physicochemical properties of wheat starch/flour modified by heat-moisture treatment. J. Cereal Sci..

[27] Blanco A., Colasuonno P., Gadaleta A., Mangini G., Schiavulli A., Simeone R., Digesù A.M., De Vita P., Mastrangelo A.M., Cattivelli L. (2011). Quantitative trait loci for yellow pigment concentration and individual carotenoid compounds in durum wheat. J. Cereal Sci..

[28] Brodkorb A., Egger L., Alminger M., Alvito P., Assunção R., Ballance S., Bohn T., Bourlieu-Lacanal C., Boutrou R., Carrière F. (2019). INFOGEST static in vitro simulation of gastrointestinal food digestion. Nat. Protoc..

[29] Lavoignat M., Juhász A., Bose U., Sayd T., Chambon C., Ribeiro M., Igrejas G., Déjean S., Ravel C., Bancel E. (2024). Peptidomics analysis of in vitro digested wheat breads: Effect of genotype and environment on protein digestibility and release of celiac disease and wheat allergy related epitopes. Food Chem..

[30] Ayadi S., Jallouli S., Chamekh Z., Zouari I., Landi S., Hammami Z., Ben Azaiez F.E., Baraket M., Esposito S., Trifa Y. (2022). Variation of Grain Yield, Grain Protein Content and Nitrogen Use Efficiency Components under Different Nitrogen Rates in Mediterranean Durum Wheat Genotypes. Agriculture.

[31] Sułek A., Cacak-Pietrzak G., Różewicz M., Nieróbca A., Grabiński J., Studnicki M., Sujka K., Dziki D. (2023). Effect of production technology intensity on the grain yield, protein content and amino acid profile in common and durum wheat grain. Plants.

[32] Siddiqi R.A., Singh T.P., Rani M., Sogi D.S., Bhat M.A. (2020). Diversity in grain, flour, amino acid composition, protein profiling, and proportion of total flour proteins of different wheat cultivars of North India. Front. Nutr..

[33] Anjun F.M., Warker C.E. (2000). Grain, flour and bread-making properties of eightPakistani hard white spring wheat cultivars grown atthree different locations for 2 years. Int. J. Food Sci. Technol..

[34] Ceseviciene J., Slepetiene A., Leistrumaite A., Ruzgas V., Slepetys J. (2012). Effects of organic and conventional production systems and cultivars on the technological properties of winter wheat. J. Sci. Food Agric..

[35] España-Fariñas M.P., Camba-Carrión J., García-Gómez M.B., Vázquez-Oderriz M.L., Lombardero-Fernanndez M., Pereira-Lorenzo S., Urquijo-Zamora L., Cobos A., Díaz O., Romero-Rodríguez M.A. (2025). Influence of cultivation system and proportion of local cultivars ‘Caaveiro’ and ‘Callobre’ in flour mixtures on the nutritional quality of Galician bread. Foods.

[36] Fernández-Canto M.N., García-Gómez M.B., Boado-Crego S., Vázquez-Odériz M.L., Muñoz-Ferreiro M.N., Lombardero Fernández M., Pereira-Lorenzo S., Romero-Rodríguez M.Á. (2022). Element content in different wheat flours and bread varieties. Foods.

[37] Vrček I.V., Čepo D.V., Rašić D., Peraica M., Žuntar I., Bojić M., Mendaš G., Medić-Šarić M. (2014). A comparison of the nutritional value and food safety of organically and conventionally produced wheat flours. Food Chem..

[38] Rembiałkowska E. (2007). Quality of plant products from organic agriculture. J. Sci. Food Agric..

[39] Le Gouis J. (2012). Quels caractères et quels outils pour améliorer l’efficacité d’utilisation de l’azote par le blé tendre?. Le Sélectionneur Français.

[40] Cabas-Lühmann P., Arriagada O., Matus I., Marcotuli I., Gadaleta A., Schwember A.R. (2023). Comparison of durum with ancient tetraploid wheats from an agronomical, chemical, nutritional, and genetic standpoints: A review. Euphytica.

[41] Agapie A.L., Horablaga M.N., Gorinoiu G., Horablaga A., Herbei M.V., Sala F. (2025). Variation of protein and protein fraction content in wheat in relation to NPK mineral fertilization. Agronomy.

[42] Zhao Z., Li Q., Xia F., Zhang P., Hao S., Sun S., Cui C., Zhang Y. (2025). Optimized water management strategies: Evaluating limited-irrigation effects on spring wheat productivity and grain nutritional composition in arid agroecosystems. Agriculture.

[43] Cai J., Zang F., Xin L., Zhou Q., Wang X., Zhong Y., Huang M., Dai T., Jiang D. (2022). Effects of cysteine and inorganic sulfur applications at different growth stages on grain protein and end-use quality in wheat. Foods.

[44] Beshah Y.B., Pescactore A., Guerrini L., Vivoli R. (2026). Sulphur and selenium foliar fertilization enhances the protein profile and reduces anti-nutritional compounds in common wheat. Field Crops Res..

[45] Punia N.D., Khetarpaul N. (2008). Physico-chemical characteristics, nutrient composition and consumer acceptability of wheat varieties grown under organic and inorganic farming conditions. Int. J. Food Sci. Nutr..

[46] Horvat D., Šimić G., Dvojković K., Ivić M., Plavšin I., Novoselović D. (2021). Gluten protein compositional changes in response to nitrogen application rate. Agronomy.

[47] Liu D., Yang H., Zhang Z., Chen Q., Guo W., Rossi V., Xin M., Du J., Hu Z., Liu J. (2023). An elite γ-gliadin allele improves end-use quality in wheat. New Phytol..

[48] Mitura K., Cacak-Pierzak G., Feledyn-Szewczyk B., Szablewski T., Studnicki M. (2023). Yield and grain quality of common wheat (*Triticum aestivum* L.) depending on the different farming systems (organic vs. integrated vs. conventional). Plants.

[49] Natale C., Galassi E., Nocente F., Taddei F., Folloni S., Visioli G., Ceccarelli S., Galaverna G., Gazza L. (2025). Rheological, technological, and nutritional profile of sustainable crops: Bread wheat evolutionary populations. Foods.

[50] Rani M., Siddiqi R.A., Singh G., Kumar R., Sogi D.S., Gill B.S. (2012). Comparative evaluation of gliadins from four extraction protocols using advanced analytical techniques. Int. J. Biol. Macromol..

[51] Singh S.K., Singhal S., Jaiswal P., Basu U., Sahi A.N., Singh A.M. (2024). Physico-chemical and rheological trait-based identification of Indian wheat varieties suitable for different end-uses. Foods.

[52] Takač V., Tóth V., Rakszegi M., Mikó P., Mikić S., Mirosavljević M. (2022). The influence of farming systems, genotype and their interaction on bioactive compound, protein and starch content of bread and spelt wheat. Foods.

[53] Day L., Augustin M.A., Batey I.L., Wrigley C.W. (2006). Wheat-gluten uses and industry needs. Trends Food Sci. Technol..

[54] Wang B., Kang J., Wang S., Haider F.U., Zhong Y., Zhang P. (2025). Variations in the end-use quality of whole grain flour are closely related to the metabolites in the grains of pigmented wheat (*Triticum aestivum* L.). Plants.

[55] Zhang R., Yang Y., Liu Q., Xu L., Bao H., Ren X., Jin Z., Jiao A. (2023). Effect of wheat gluten and peanut protein ratio on the moisture distribution and textural quality of high moisture extruded meat analogs from an extruder response perspective. Foods.

[56] Šekularac A., Torbica A., Živančev D., Tomi’c J., Kneževi’c D. (2018). The influence of wheat genotype and environmental factors on gluten index and the possibility of its use as bread quality predictor. Genetika.

[57] Kwiatkowski C.A., Haliniarz M., Tomczyńska-Mleko M., Mleko S., Kawecka-Radomska M. (2015). The content of dietary fiber, amino acids, dihydroxyphenols and some macro- and micronutrients in grain of conventionally and organically grown common wheat, spelt wheat and proso millet. Agric. Food Sci..

[58] Zhang P., Ma G., Wang C., Lu H., Li S., Xie Y., Ma D., Zhu Y., Guo T. (2017). Effect of irrigation and nitrogen application on grain amino acid composition and protein quality in winter wheat. PLoS ONE.

[59] Cormier F., Faure S., Dubreuil P., Heumez E., Beauchene K., Lafarge S., Praud S., Le Gouis J. (2013). A multi-enviornmental study of recent breeding progress on nitrogen use efficiency in wheat (*Triticum aestivum* L.). Theor. Appl. Genet..

[60] Yang Q., Zhao D., Liu Q. (2020). Connections between amino acid metabolisms in plants: Lysine as an example. Front. Plant Sci..

[61] Ran J., Tang Y., Zhang Y., Jiao L., Zhang C., Li Y., Zhao R. (2024). Mixed fermentation of lactic acid bacteria and sourdough on quality and storage characteristics of steamed bun. Food Chem. X.

[62] Gorinoiu G., Petolescu C., Agapie A.L., Buzna C., Rain P., Horablaga N.M., Horablaga A., Samfira I., Boldea M.V., Petrescu I. (2025). Yeld, protein, and starch equilibrium of indigenous varieties: An open door for computational breeding in enhancing selection strategies. Agronomy.

[63] Brandt K., Mølgaard J.P. (2001). Organic agriculture, does it enhance or reduce thenutritional value of plant foods?. J. Sci. Food Agric..

[64] Wang S., Copeland L. (2013). Molecular disassembly of starch granules during gelatinization and its effect on starch digestibility: A review. Food Funct..

[65] Li F., Zhao C., Xu D., Cheng D., Liu A., Zhai S., Zi Y., Cao X., Liu C., Song J. (2025). Understanding the formation of starch structure during grain development in two wheat cultivars with different filling rate. Carbohydr. Polym..

[66] Li G., Hu Q., Shi Y., Cui K., Nie L., Huang J., Peng S. (2018). Low nitrogen application enhances starch-metabolizing enzyme activity and improves accumulation and translocation of non-structural carbohydrates in rice stems. Front. Plant Sci..

[67] Li H., Liu Y. (2019). Effects of variety and growth location on the chain-length distribution of rice starches. J. Cereal Sci..

[68] Nhan M.T., Copeland L. (2016). Effect of variety and growing environment on pasting and thermal properties of wheat starch. Starch-Stärke.

[69] De Arcangelis E., Trivisonno M.C., Angelicola M., Quiquero M., Di Nardo V., Falasca L., Sestili F., Messia M.C., Marconi E. (2021). Milling and rheological properties of high amylose wheat. J. Cereal Sci..

[70] Jia B., Devkota L., Dhital S. (2025). Protein structure and functional differentiation between high-amylose and wild-type wheat. Food Hydrocoll..

[71] Moon Y., Kweon M. (2025). Processing Suitability of Physical Modified Non-GMO High-Amylose Wheat Flour as a Resistant Starch Ingredient in Cookies. Molecules.

[72] Seyedain-Ardabili M., Azizi M.H., Salami M. (2023). Evaluation of antioxidant, α-amylase-inhibitory and antimicrobial activities of wheat gluten hydrolysates produced by ficin protease. J. Food Meas. Charact..

[73] Guo R., Liu L., Huang Y., Lv M., Zhu Y., Wang Z., Zhu X., Sun B. (2023). Effect of Na^+^ and Ca^2+^ on the texture, structure and microstructure of composite protein gel of mung bean protein and wheat gluten. Food Res. Int..

[74] Žilić S., Barać M., Pešić M., Dodig D., Ignjatović-Micić D. (2011). Characterization of proteins from grain of different bread and durum wheat genotypes. Int. J. Mol. Sci..

[75] Mandal S., Verma A.K. (2021). Wheat breeding, fertilizers, and pesticides: Do they contribute to the increasing immunogenic properties of modern wheat?. Gastrointest. Disord..

[76] Boukid F., Prandi B., Sforza S., Sayar R., Seo Y.W., Mejri M., Yacoubi I. (2017). Understanding the effects of genotype, growing year, and breeding on Tunisian durum wheat allergenicity. 2. The celiac disease case. J. Agric. Food Chem..

[77] Ronga D., Laviano L., Catellani M., Milc J., Prandi B., Boukid F., Sforza S., Dossena A., Graziano S., Gullì M. (2020). Influence of environmental and genetic factors on content of toxic and immunogenic wheat gluten peptides. Eur. J. Agron..

[78] Larré C., Lupi R., Gombaud G., Brossard C., Branlard G., Moneret-Vautrin D.A., Rogniaux H., Denery-Papini S. (2011). Assessment of allergenicity of diploid and hexaploid wheat genotypes: Identification of allergens in the albumin/globulin fraction. J. Proteom..

[79] Lexhaller B., Colgrave M.L., Scherf K.A. (2019). Characterization and relative quantitation of wheat, rye, and barley gluten protein types by liquid chromatography–tandem mass spectrometry. Front. Plant Sci..

